# Supplementary material for: Transcript-specific induction of stop codon readthrough using a CRISPR-dCas13 system
Source: EMBO Rep. 2024 Mar 18;25(4):26. doi: 10.1038/s44319-024-00115-8 (PMC11015002; doi:10.1038/s44319-024-00115-8)
Supplement: Supplementary file 1 — Appendix [file 44319_2024_115_MOESM1_ESM.pdf]

# Appendix

**Table of contents** **Page no.**


1. Appendix Figure S1 2
2. Appendix Figure S2 3
3. Appendix Figure S3 4
4. Appendix Figure S4 5
5. Appendix Figure S5 6

## Appendix Figure S1

CCGGCATGGCTGTTAGCTTGGTACC**ATG**GTGCGGGTACAGCGACCACGGCAAGAGATCATTG
AAGACTTGTCCTACATGGTGCGTGAGCTCCTCATCCAATTCTACAAGTCCACCCGTTTCAAG
CCTACCCGCATCATCTTCTACCGAGATGGGGTGCCTGAAGGCCAGCTACCCCAGATACTCCA
CTATGAGCTACTGGCCATTCGTGATGCCTGCATCAAACTGGAAAAGGACTACCAGCCTGGGA
TCACTTATATTGTGGTGCAGAAACGCCATCACACCCGCCTTTTCTGTGCTGACAAGAATGAG
CGAATTGGGAAGAGTGGTAACATCCCAGCTGGGACCACAGTGGACACCAACATCACCCACCC
ATTTGAGTTTGACTTCTATCTGTGCAGCCACGCAGGCATCCAGGGCACCAGCCGACCATCCC
ATTACTATGTTCTTTGGGATGACAACCGTTTCACAGCAGATGAGCTCCAGATCCTGACGTAC
CAGCTGTGCCACACTTACGTACGATGCACACGCTCTGTCTCTATCCCAGCACCTGCCTACTA
TGCCCGCCTGGTGGCTTTCCGGGCACGATACCACCTGGTGGACAAGGAGCATGACAGTGGAG
AGGGGAGCCACATATCGGGGCAGAGCAATGGGCGGGACCCCCAGGCCCTGGCCAAAGCCGTG
CAGGTTCACCAGGATACTCTGCGCACCATGTACTTCGCT**TGA**AGGCAGAACGCTGTTACCTC
ACTGGATAGAAGAAAGCTTTCCAAGCCCCAGGAGCTGTGCCACCCAAATCCAGAGGAAGCAA
GGAGGAGGGAGGTGGGGGGATCCGACTACAAGGACGACGATGACAAGTACCCTTATGACGTG
CCCGATTACGCTAGCGGCCGCTCGAGTCTAGAGGGCCCTTCGAACAAAAACTCATCTCAGAA
GAGGATC**TGA**ATATGCATACCGGTCATCATCACCATCACCATTGAGTTTAAACCCGCTGATC
ACCTCAGT

**Appendix Figure S1.** Result of sequencing of *AGO1*-UGA-3′UTR-*FLAG-HA* construct (see Fig 1B, C and EV2B). T7 forward primer (5′ TAATACGACTCACTATAGGG 3′) was used for sequencing. Start codon – bold and green; *AGO1* coding sequence – blue; Canonical stop codon and the stop codon after FLAG-HA tag – bold and red; proximal 3′UTR of *AGO1* – purple; FLAG tag – dark orange; HA tag – orange; pCDNA vector backbone – black.

## Appendix Figure S2

**ATG**GTGCGGGTACAGCGACCACGGCAAGAGATCATTGAAGACTTGTCCTACATGGTGCGTGA
GCTCCTCATCCAATTCTACAAGTCCACCCGTTTCAAGCCTACCCGCATCATCTTCTACCGAG
ATGGGGTGCCTGAAGGCCAGCTACCCCAGATACTCCACTATGAGCTACTGGCCATTCGTGAT
GCCTGCATCAAACTGGAAAAGGACTACCAGCCTGGGATCACTTATATTGTGGTGCAGAAACG
CCATCACACCCGCCTTTTCTGTGCTGACAAGAATGAGCGAATTGGGAAGAGTGGTAACATCC
CAGCTGGGACCACAGTGGACACCAACATCACCCACCCATTTGAGTTTGACTTCTATCTGTGC
AGCCACGCAGGCATCCAGGGCACCAGCCGACCATCCCATTACTATGTTCTTTGGGATGACAA
CCGTTTCACAGCAGATGAGCTCCAGATCCTGACGTACCAGCTGTGCCACACTTACGTACGAT
GCACACGCTCTGTCTCTATCCCAGCACCTGCCTACTATGCCCGCCTGGTGGCTTTCCGGGCA
CGATACCACCTGGTGGACAAGGAGCATGACAGTGGAGAGGGGAGCCACATATCGGGGCAGAG
CAATGGGCGGGACCCCCAGGCCCTGGCCAAAGCCGTGCAGGTTCACCAGGATACTCTGCGCA
CCATGTACTTCGCT**TGA**AGGCAGAACGCTGTTACCTCACTGGATAGAAGAAAGCTTTCCAAG
CCCCAGGAGCTGTGCCACCCAAATCCAGAGGAAGCAAGGAGGAGGGAGGTGGGGGGCGGCTC
CGGCGGCTCCCTCGTGCTCGAGGAAGACGCCAAAAACATAAAGAAAGGCCCGGCGCCATTCT
ATCCTCTAGAGGATGGAACCGCTGGAGAGCAACTGCATAAGGCTATGAAGAGATACGCCCTG
GTTCCTGGAACAATTGCTTTTACAGATGCACATATCGAGGTGAACATCACGTACGCGGAATA
CTTCGAAATGTCCGTTCGGTTGGCAGAAGCTATGAAACGATATGGGCTGAATACAAATCACA
GAATCGTCGTATGCAGTGAAAACTCTCTTCAATTCTTTATGCCGGTGTTGGGCGCGTTATTT
ATCGGAGTTGCAGTTGCGCCCGCGAACGACATTTATAATGAACGTGAATTGCTCAACAGTAT
GAACATTTCGCAGCCTACCGTAGTGTTTGTTTCCAAAAAGGGGTTGCAAAAAATTTTGAACG
TGCAAAAAAAATTACCAATAATCCAGAAAATTATTATCATGGATTCTAAAACGGATTACCAG
GGATTTCAGTCGATGTACACGTTCGTCACATCTCATCTACCTCCCGGTTTTAATGAATACGA
TTTTGTACCAGAGTCCTTTGATCGTGACAAAACAATTGCACTGATAATGAATTCCTCTGGAT
CTACTGGGTTACCTAAGGGTGTGGCCCTTCCGCATAGAACTGCCTGCGTCAGATTCTCGCAT
GCCAGAGATCCTATTTTTGGCAATCAAATCATTCCGGATACTGCGATTTTAAGTGTTGTTCC
ATTCCATCACGGTTTTGGAATGTTTACTACACTCGGATATTTGATATGTGGATTTCGAGTCG
TCTTAATGTATAGATTTGAAGAAGAGCTGTTTTTACGATCCCTTCAGGATTACAAAATTCAA
AGTGCGTTGCTAGTACCAACCCTATTTTCATTCTTCGCCAAAAGCACTCTGATTGACAAATA
CGATTTATCTAATTTACACGAAATTGCTTCTGGGGGCGCACCTCTTTCGAAAGAAGTCGGGG
AAGCGGTTGCAAAACGCTTCCATCTTCCAGGGATACGACAAGGATATGGGCTCACTGAGACT
ACATCAGCTATTCTGATTACACCCGAGGGGGATGATAAACCGGGCGCGGTCGGTAAAGTTGT
TCCATTTTTTGAAGCGAAGGTTGTGGATCTGGATACCGGGAAAACGCTGGGCGTTAATCAGA
GAGGCGAATTATGTGTCAGAGGACCTATGATTATGTCCGGTTATGTAAACAATCCGGAAGCG
ACCAACGCCTTGATTGACAAGGATGGATGGCTACATTCTGGAGACATAGCTTACTGGGACGA
AGACGAACACTTCTTCATAGTTGACCGCTTGAAGTCTTTAATTAAATACAAAGGATATCAGG
TGGCCCCCGCTGAATTGGAATCGATATTGTTACAACACCCCAACATCTTCGACGCGGGCGTG
GCAGGTCTTCCCGACGATGACGCCGGTGAACTTCCCGCCGCCGTTGTTGTTTTGGAGCACGG
AAAGACGATGACGGAAAAAGAGATCGTGGATTACGTCGCCAGTCAAGTAACAACCGCGAAAA
AGTTGCGCGGAGGAGTTGTGTTTGTGGACGAAGTACCGAAAGGTCTTACCGGAAAACTCGAC
GCAAGAAAAATCAGAGAGATCCTCATAAAGGCCAAGAAGGGCGGAAAGTCCAAATTG**TAA**

**Appendix Figure S2.** Result of sequencing of *AGO1*-UGA-3′UTR-*FLuc* construct (see Fig 1E, EV1E, and EV2C). Multiple primers were used for sequencing. Start codon – bold and green; *AGO1* coding sequence – blue; Canonical stop codon and the stop codon after firefly luciferase (FLuc) – bold and red; proximal 3′UTR of *AGO1* – purple; linker sequence – orange; FLuc – brown. Following primers (5′ to 3′) were used for sequencing: *AGO1* F1- GTGCGGG TACA GCGACCACGGCAAGAG; *AGO1* F2- GGGAGCCACATATCGGGGCAG; FLuc forward - GAAGACGCCAAAAACATA; FLuc reverse - TTACAATTTGGACTTTCCG; BGH Reverse - TAGAAGGCACAGTCGAGG.

## Appendix Figure S3

```
                                                       WT CAAAG CCGTGCAGGT TCACCAGGAT
                                                       Δ  CCAAG CCGTGCAGGT TCACCAGGAT
                                                          *  *** ********** **********

WT ACTCTGCGCA CCATGTACTT CGCTTGAAGG CAGAACGCTG TTACCTCACT GGATAGAAGA
Δ  ACTCTGCGCA CCATGTACCT TGCTTGAAGG CAGAACGCTG TTACCTC--- ----------
   ********** ******** *  ********* ********** *******

WT AAGCTTTCCA AGCCCCAGGA GCTGTGCCAC CCAAATCCAG AGGAAGCAAG GAGGAG-GGA
Δ  ---------- ---------- ---------- --------AG AGGAAGCAAG GAGGAGAGGA
                                            ** ********** ****** ***

WT GGTGGGGTAG GG…
Δ  GGGGGGGTAG GG…
   ** ******* **
```

**Appendix Figure S3.** Alignment of wild-type sequence (WT, blue) of *AGO1* region near its canonical stop codon, and the same region in HEK293 cells transfected with Cas9 and sgRNAs (Δ, brown). *, perfect match.

## Appendix Figure S4

```
                                   WT ACCCC AGCCTTTGTT TTCCATTTCC
                                   Δ  ACCCC AGCCTTTGTT TTCCATTTCC
                                      ***** ********** **********

WT CTCAGATGTG ACAAGCCGAG GCGGTGAGCC GGGCAGGAGG AAGGAGCCTC CCTCAGGGTT
Δ  CTCAGATGTG ACAAGCCGAG GCGGTGAGCC GGGCAGGAGG AAGGAGCCTC C---------
   ********** ********** ********** ********** ********** *

WT TCGGGAACCA GATCTCTCAC CAGGAAAGAC TGATACAGAA CGATCGATAC AGAAACCACG
Δ  ---------- ---------- ---------- ---------- ---------- ----------


WT CTGCCGCCAC CACACCATCA CCATCGACAG AACAGTCCTT AATCCAGAAA CC…
Δ  ---------- ---------- ------ACAG AACAGTCCTT AATCCAGAAA CC…
                                **** ********** ********** **
```

**Appendix Figure S4.** Alignment of wild-type sequence (WT, blue) of *VEGFA* region near its canonical stop codon, and the same region in HEK293 cells transfected with Cas9 and sgRNAs(Δ, brown). *, perfect match.

## Appendix Figure S5

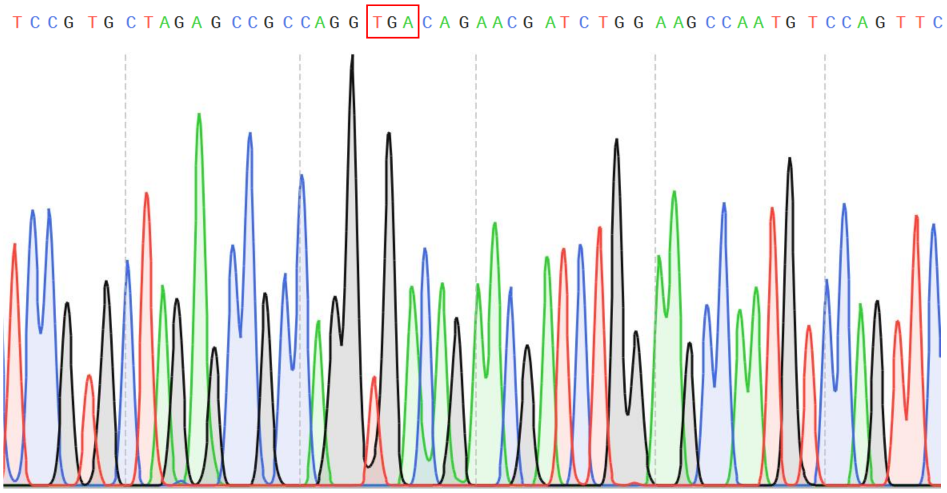


**Appendix Figure S5.** Electropherogram showing the PTC (TGA, red box) generated using CRISPR-Cas9 system at 891st codon of *SPTA1* gene in K562 cells.
