# Supplementary figures and images for: Transcript-specific induction of stop codon readthrough using a CRISPR-dCas13 system
Source: EMBO Rep. 2024 Mar 18;25(4):26. doi: 10.1038/s44319-024-00115-8 (PMC11015002; doi:10.1038/s44319-024-00115-8)

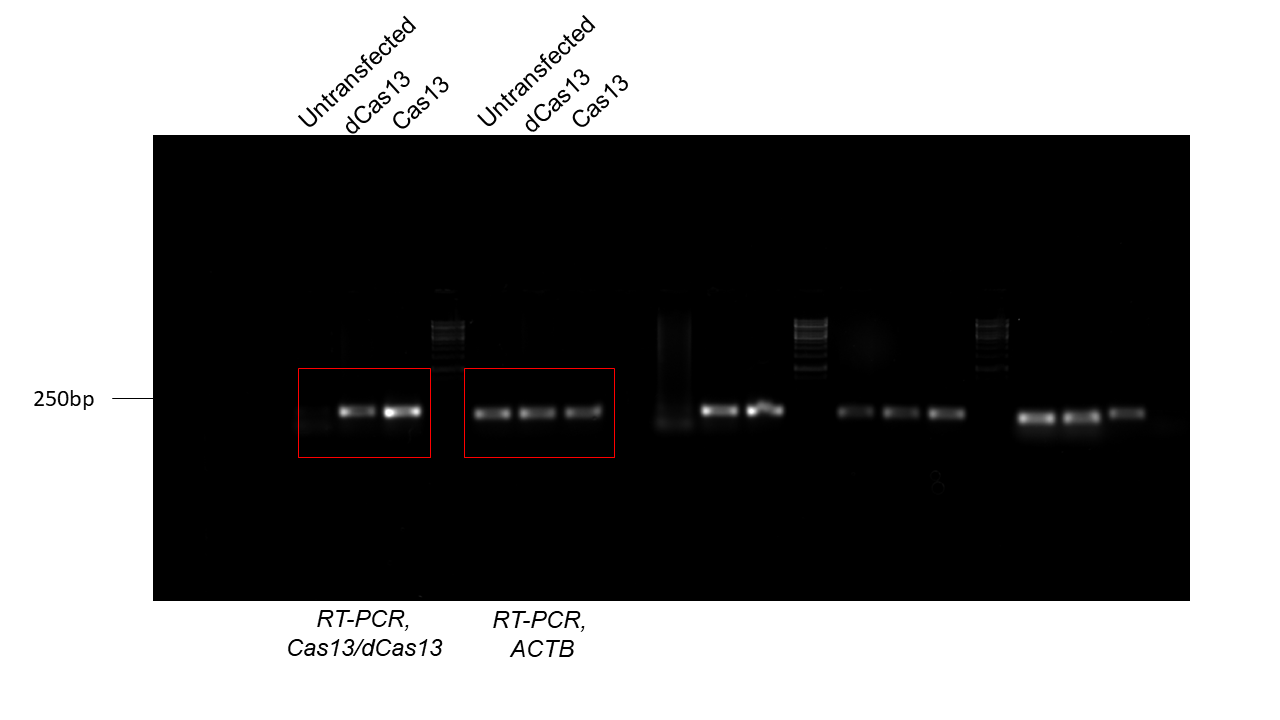

Supplement: Supplementary file 2 — Figure EV1 to EV5 Source Data [file 44319_2024_115_MOESM2_ESM.zip › EV Figures/Figure EV1/Figure EV1A/RT-PCR.TIF]

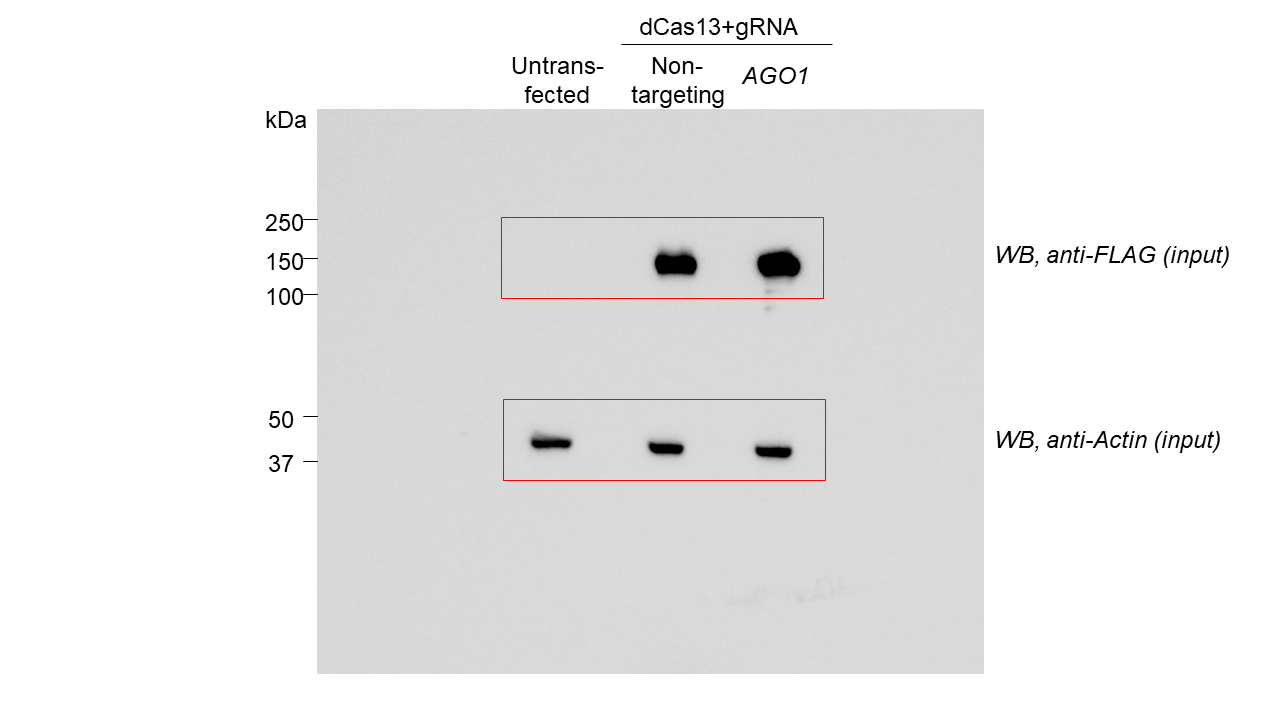

Supplement: Supplementary file 2 — Figure EV1 to EV5 Source Data [file 44319_2024_115_MOESM2_ESM.zip › EV Figures/Figure EV1/Figure EV1B/WB, input.TIF]

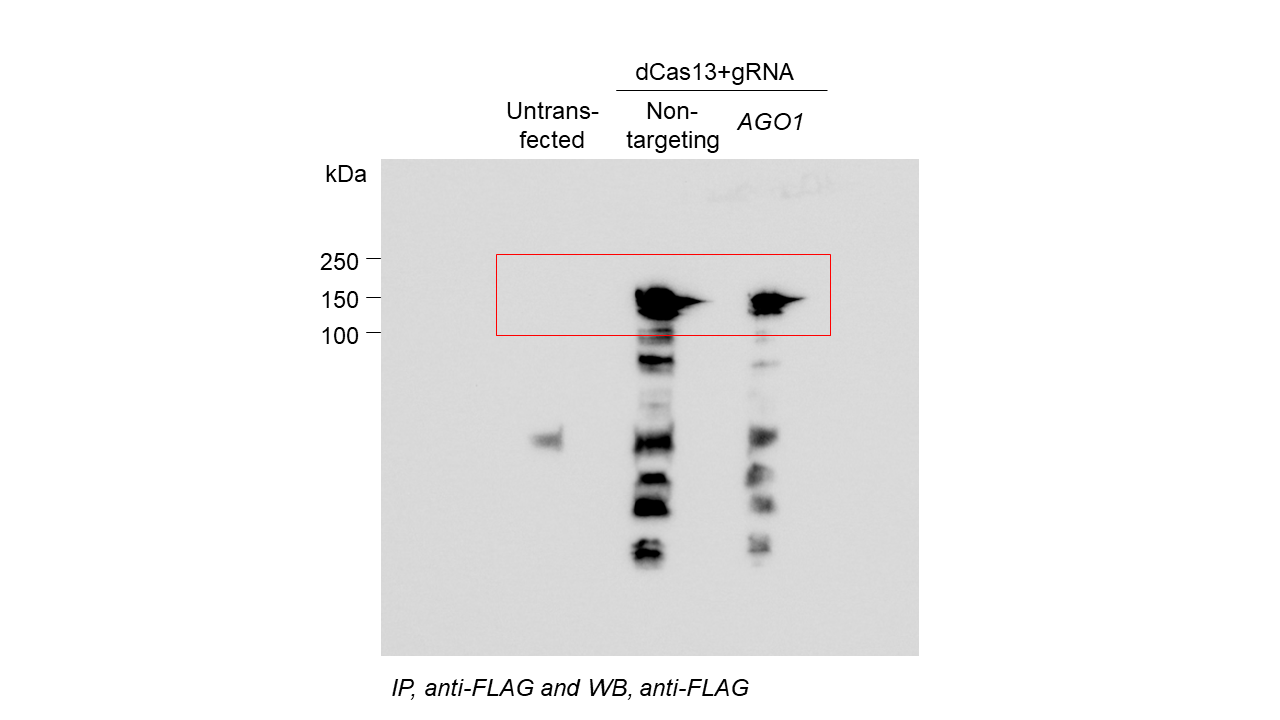

Supplement: Supplementary file 2 — Figure EV1 to EV5 Source Data [file 44319_2024_115_MOESM2_ESM.zip › EV Figures/Figure EV1/Figure EV1B/WB, IP.TIF]

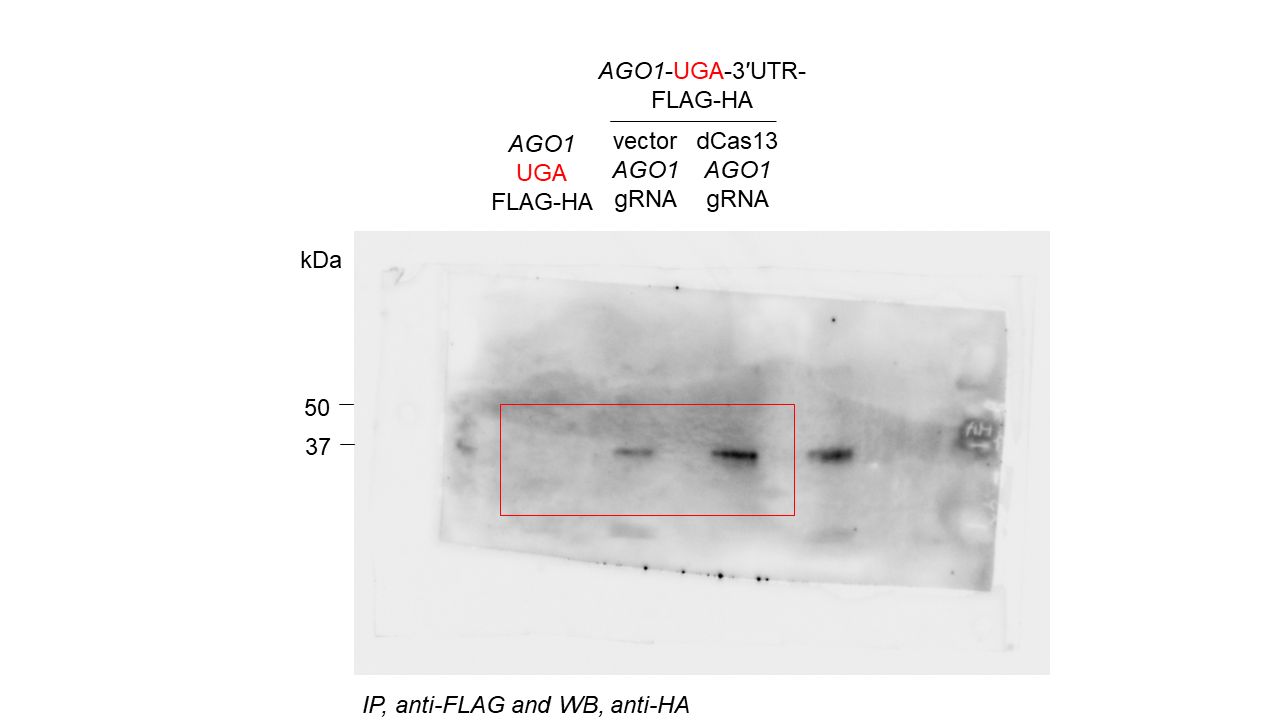

Supplement: Supplementary file 2 — Figure EV1 to EV5 Source Data [file 44319_2024_115_MOESM2_ESM.zip › EV Figures/Figure EV1/Figure EV1D/IP, anti-FLAG and WB, anti-HA.TIF]

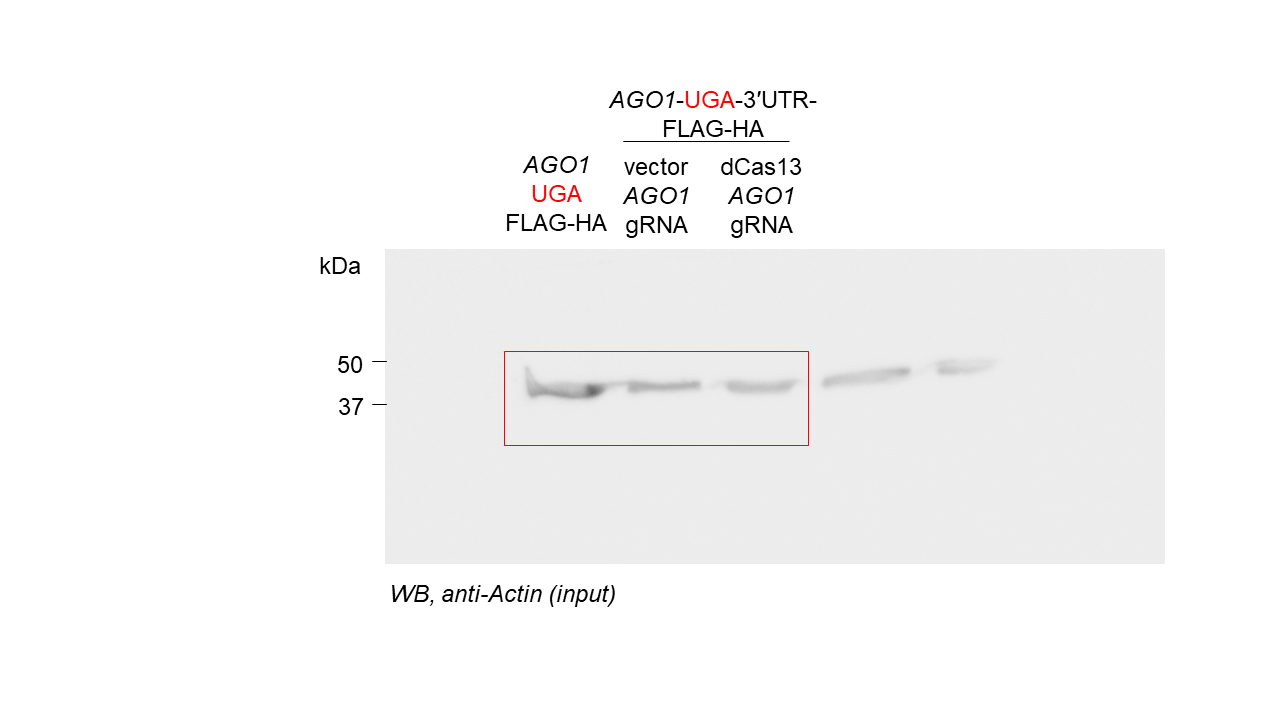

Supplement: Supplementary file 2 — Figure EV1 to EV5 Source Data [file 44319_2024_115_MOESM2_ESM.zip › EV Figures/Figure EV1/Figure EV1D/WB, anti-Actin (Input).TIF]

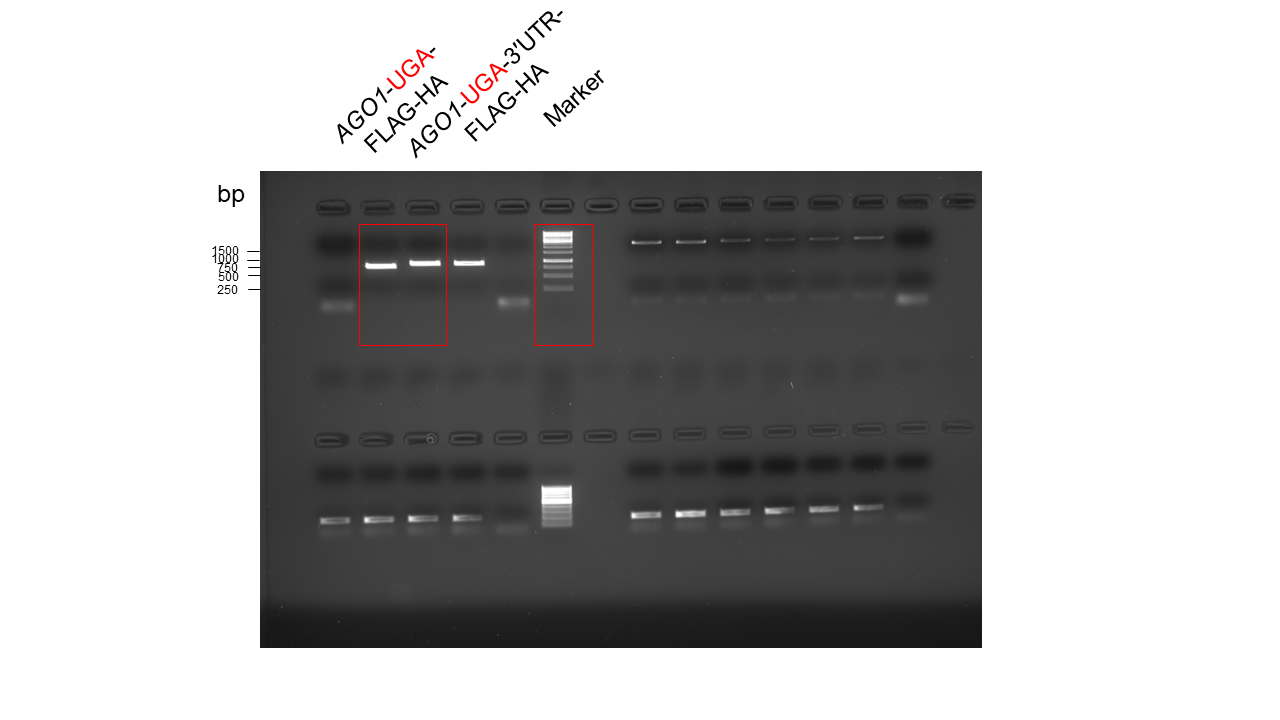

Supplement: Supplementary file 2 — Figure EV1 to EV5 Source Data [file 44319_2024_115_MOESM2_ESM.zip › EV Figures/Figure EV2/Figure EV2B/RT-PCR.TIF]

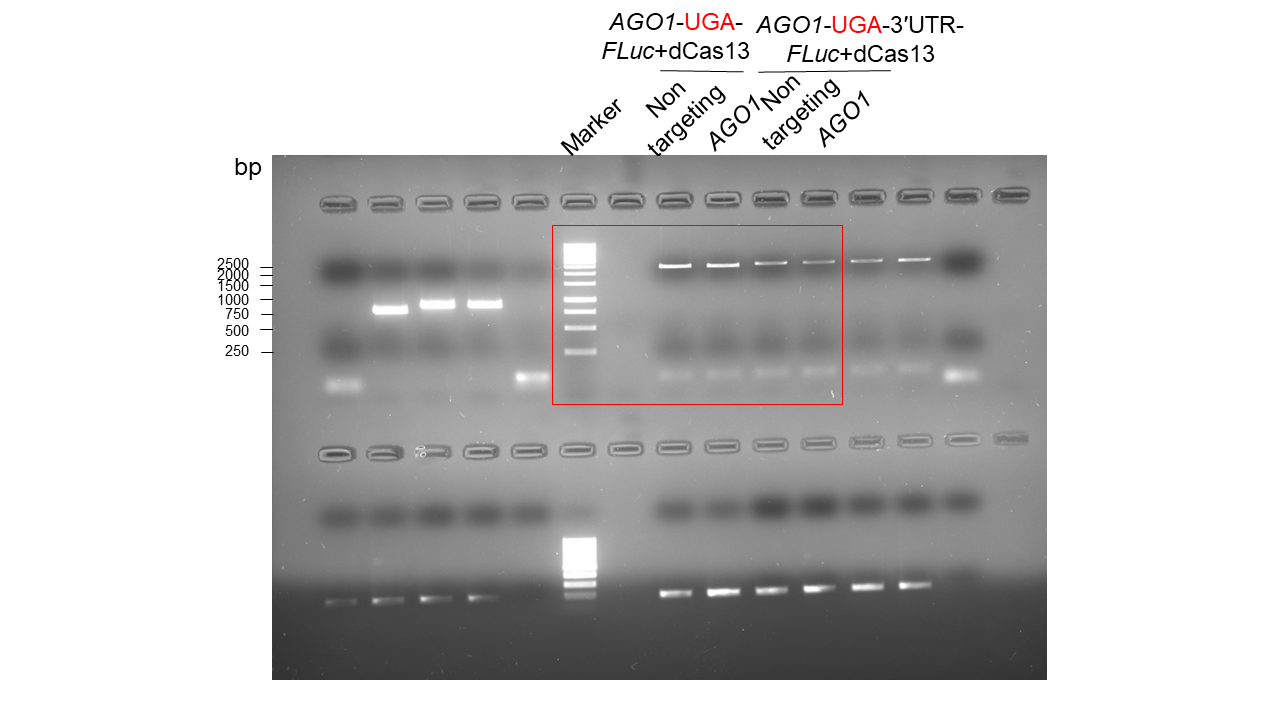

Supplement: Supplementary file 2 — Figure EV1 to EV5 Source Data [file 44319_2024_115_MOESM2_ESM.zip › EV Figures/Figure EV2/Figure EV2C/RT-PCR.TIF]

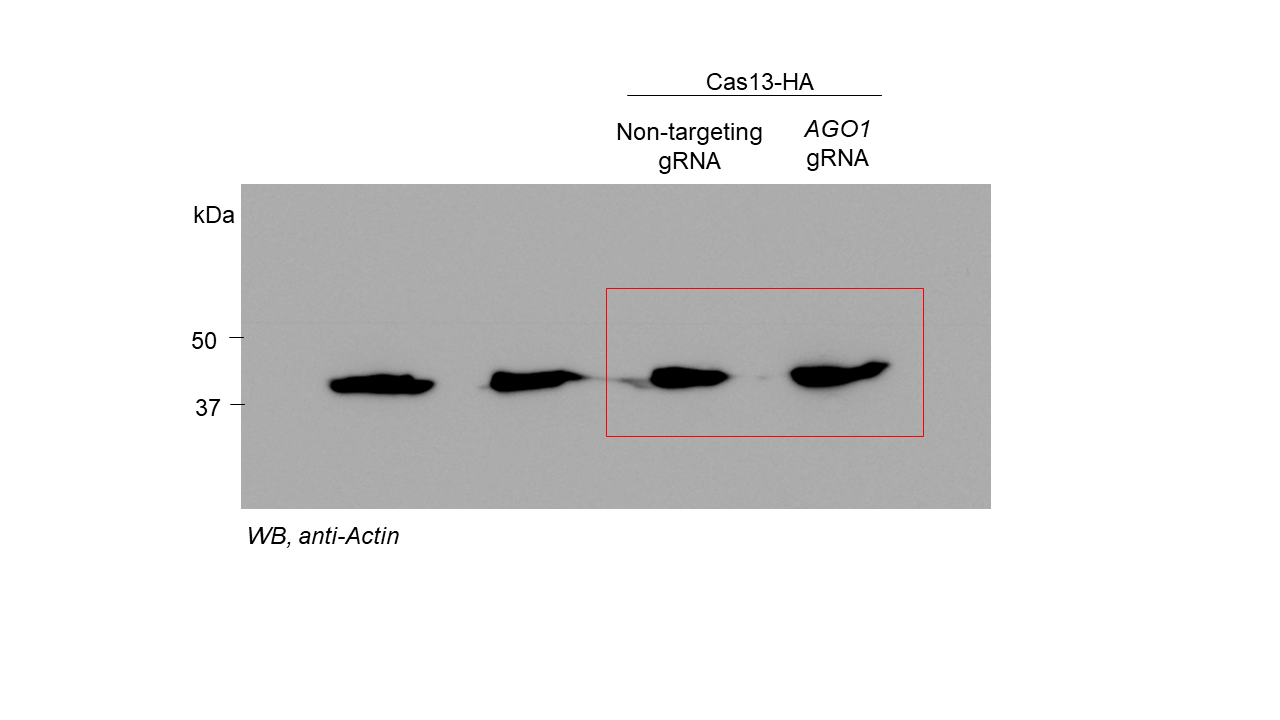

Supplement: Supplementary file 2 — Figure EV1 to EV5 Source Data [file 44319_2024_115_MOESM2_ESM.zip › EV Figures/Figure EV3/Figure EV3A/WB, anti-Actin.TIF]

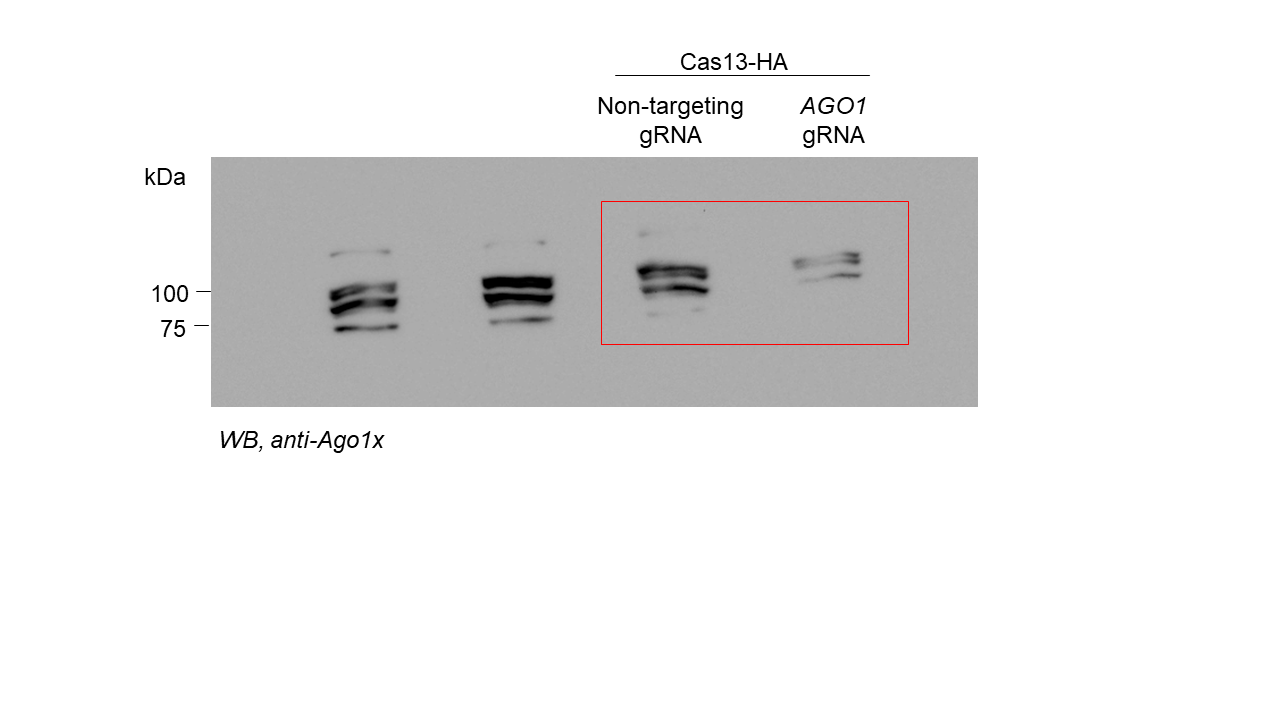

Supplement: Supplementary file 2 — Figure EV1 to EV5 Source Data [file 44319_2024_115_MOESM2_ESM.zip › EV Figures/Figure EV3/Figure EV3A/WB, anti-Ago1x.TIF]

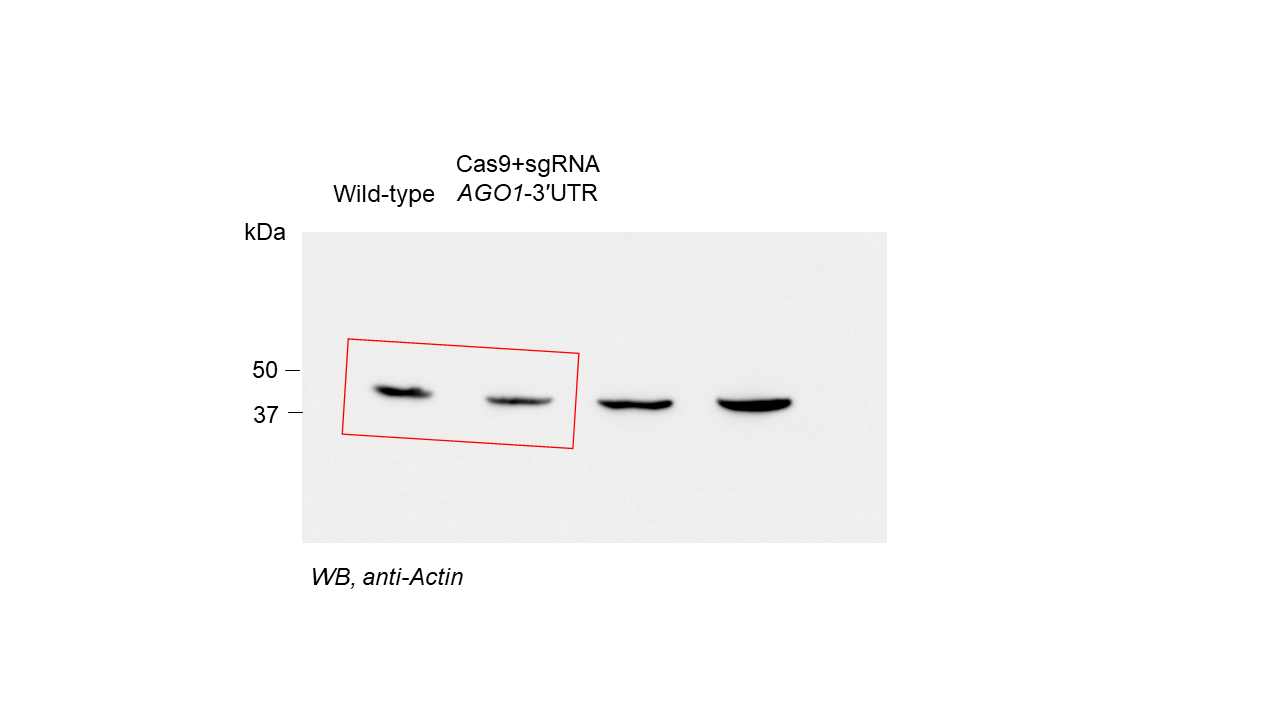

Supplement: Supplementary file 2 — Figure EV1 to EV5 Source Data [file 44319_2024_115_MOESM2_ESM.zip › EV Figures/Figure EV3/Figure EV3B/WB, anti-Actin.TIF]

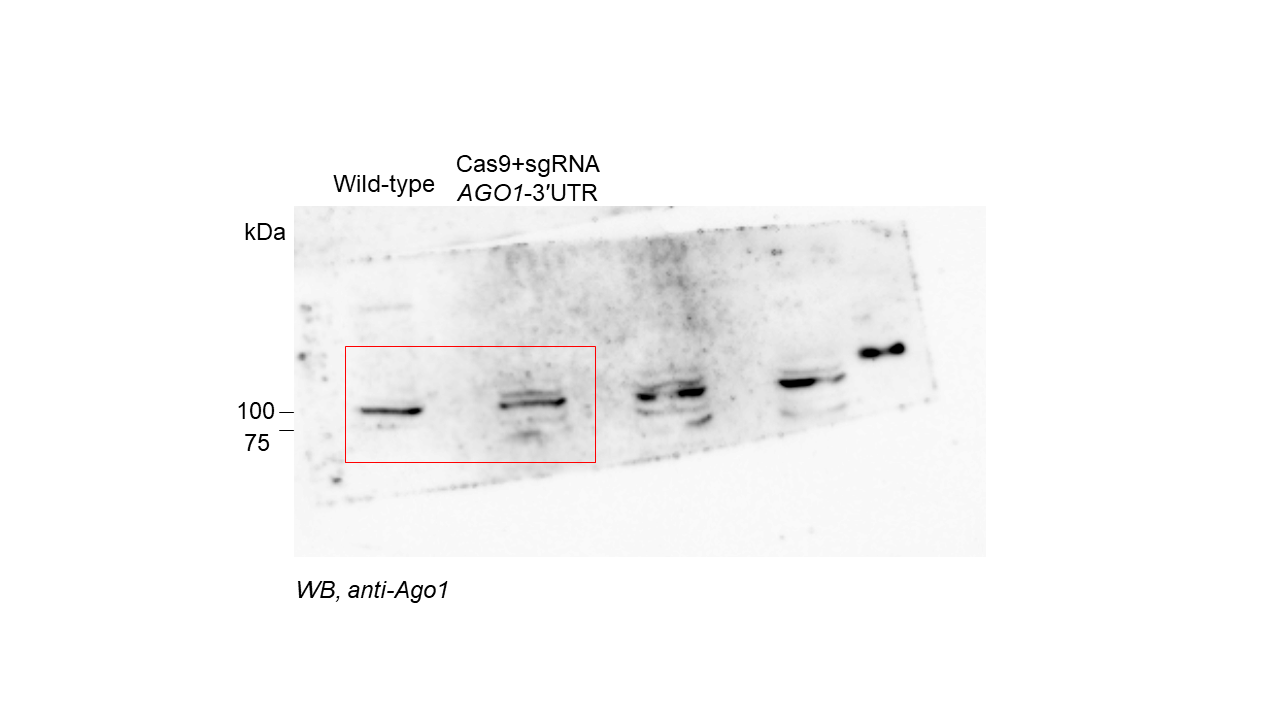

Supplement: Supplementary file 2 — Figure EV1 to EV5 Source Data [file 44319_2024_115_MOESM2_ESM.zip › EV Figures/Figure EV3/Figure EV3B/WB, anti-Ago1.TIF]

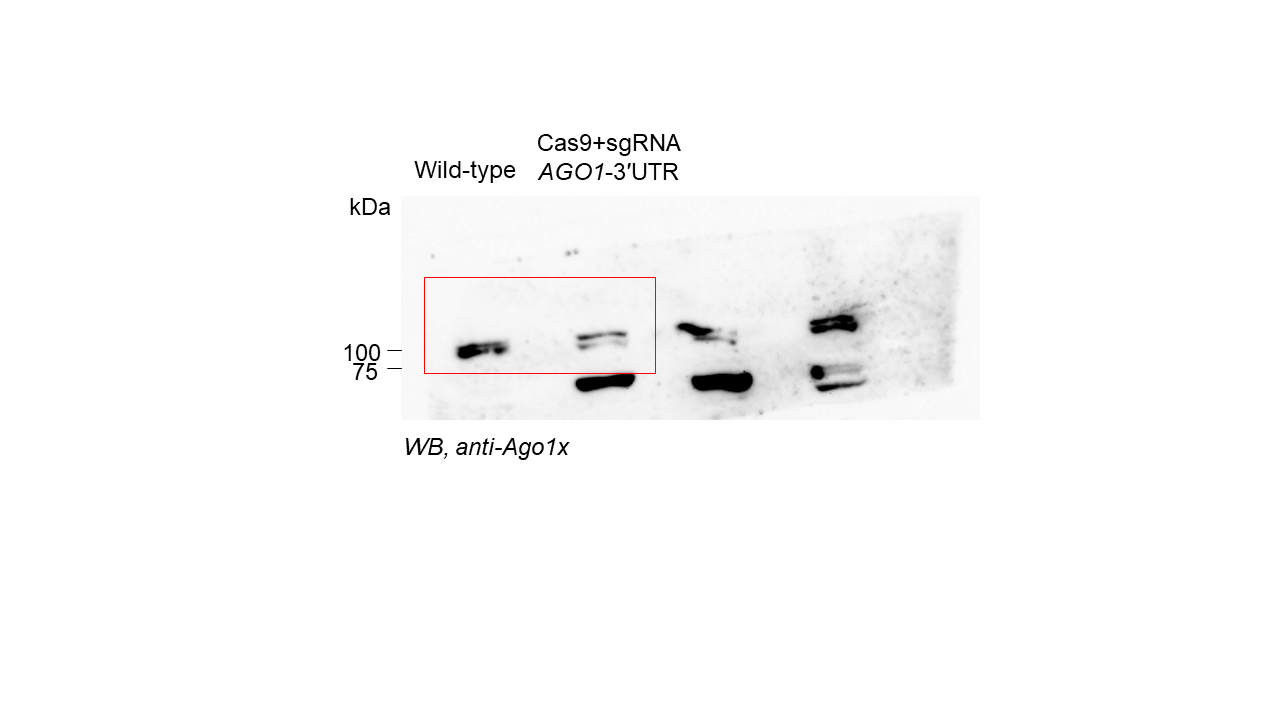

Supplement: Supplementary file 2 — Figure EV1 to EV5 Source Data [file 44319_2024_115_MOESM2_ESM.zip › EV Figures/Figure EV3/Figure EV3B/WB, anti-Ago1x.TIF]

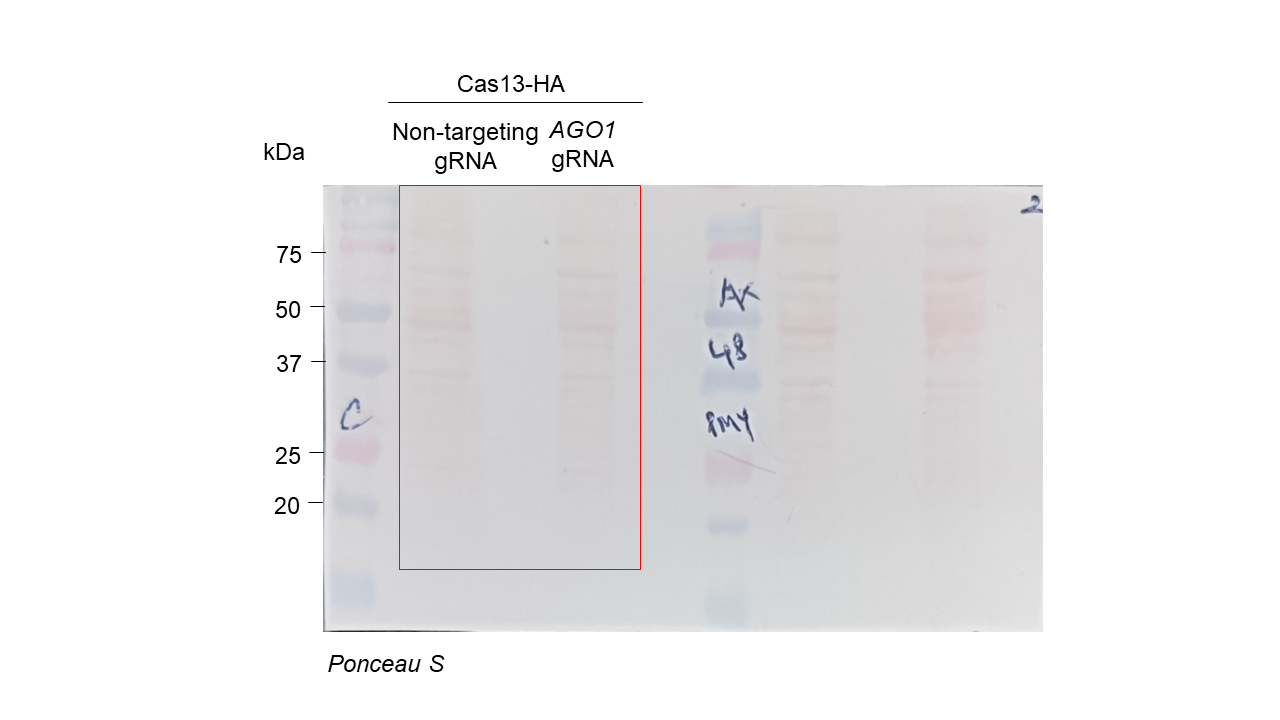

Supplement: Supplementary file 2 — Figure EV1 to EV5 Source Data [file 44319_2024_115_MOESM2_ESM.zip › EV Figures/Figure EV3/Figure EV3C/Ponceau S.TIF]

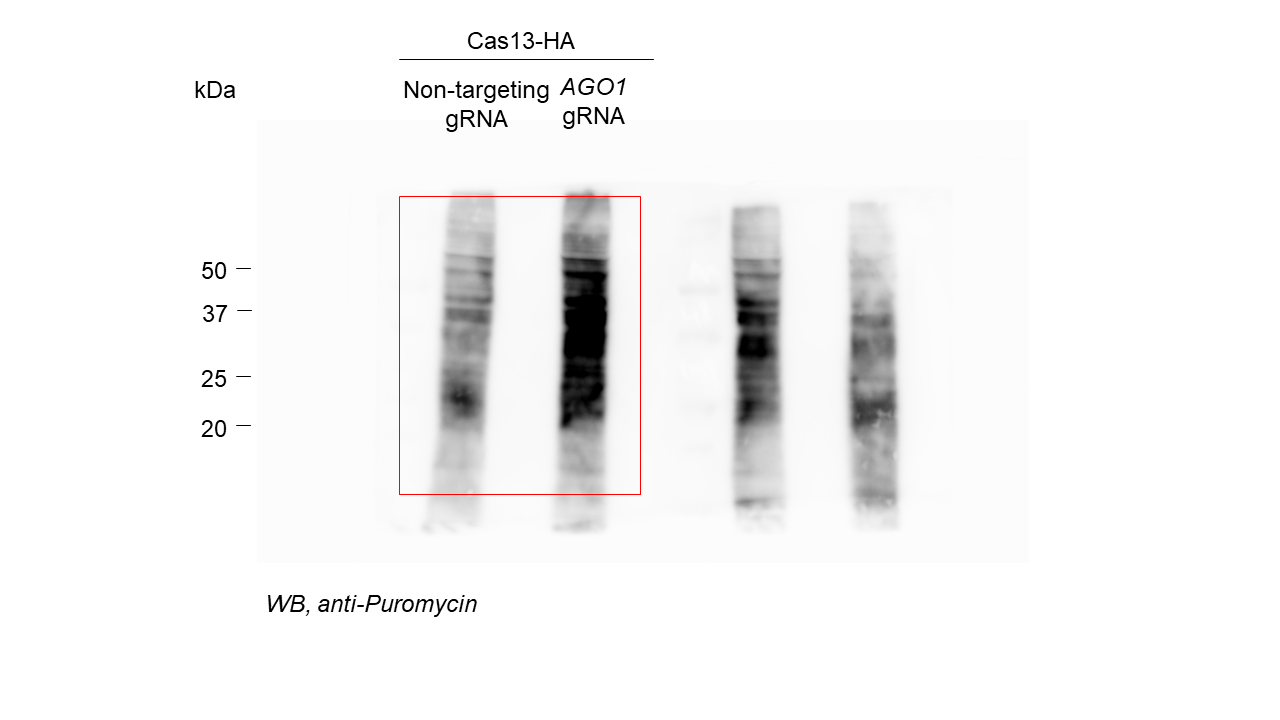

Supplement: Supplementary file 2 — Figure EV1 to EV5 Source Data [file 44319_2024_115_MOESM2_ESM.zip › EV Figures/Figure EV3/Figure EV3C/WB, anti-Puromycin.TIF]

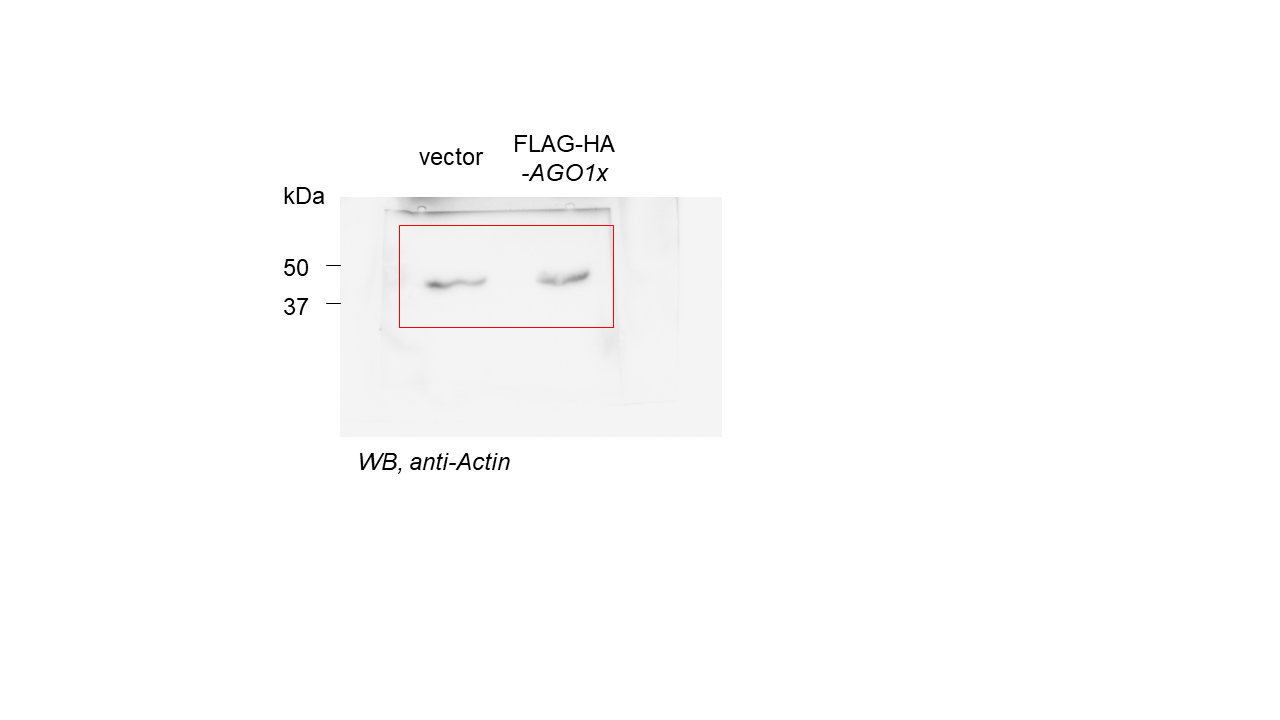

Supplement: Supplementary file 2 — Figure EV1 to EV5 Source Data [file 44319_2024_115_MOESM2_ESM.zip › EV Figures/Figure EV3/Figure EV3D/WB, anti-Actin.TIF]

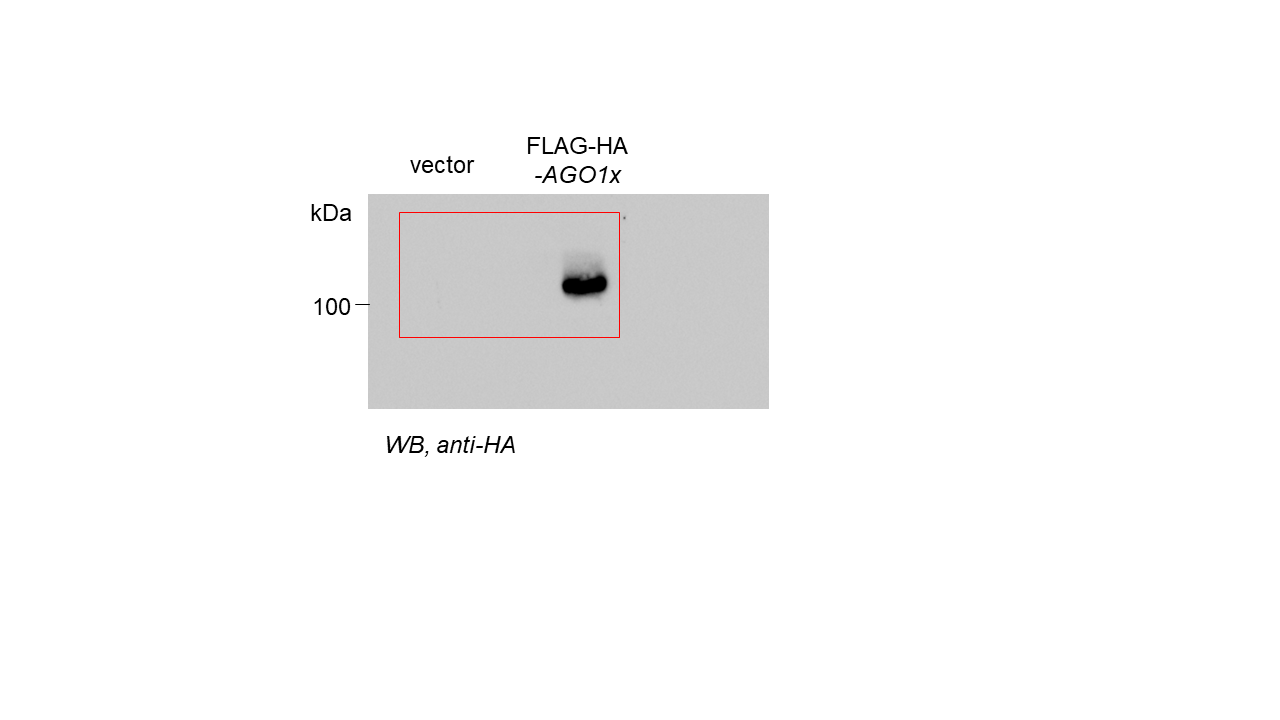

Supplement: Supplementary file 2 — Figure EV1 to EV5 Source Data [file 44319_2024_115_MOESM2_ESM.zip › EV Figures/Figure EV3/Figure EV3D/WB, anti-HA.TIF]

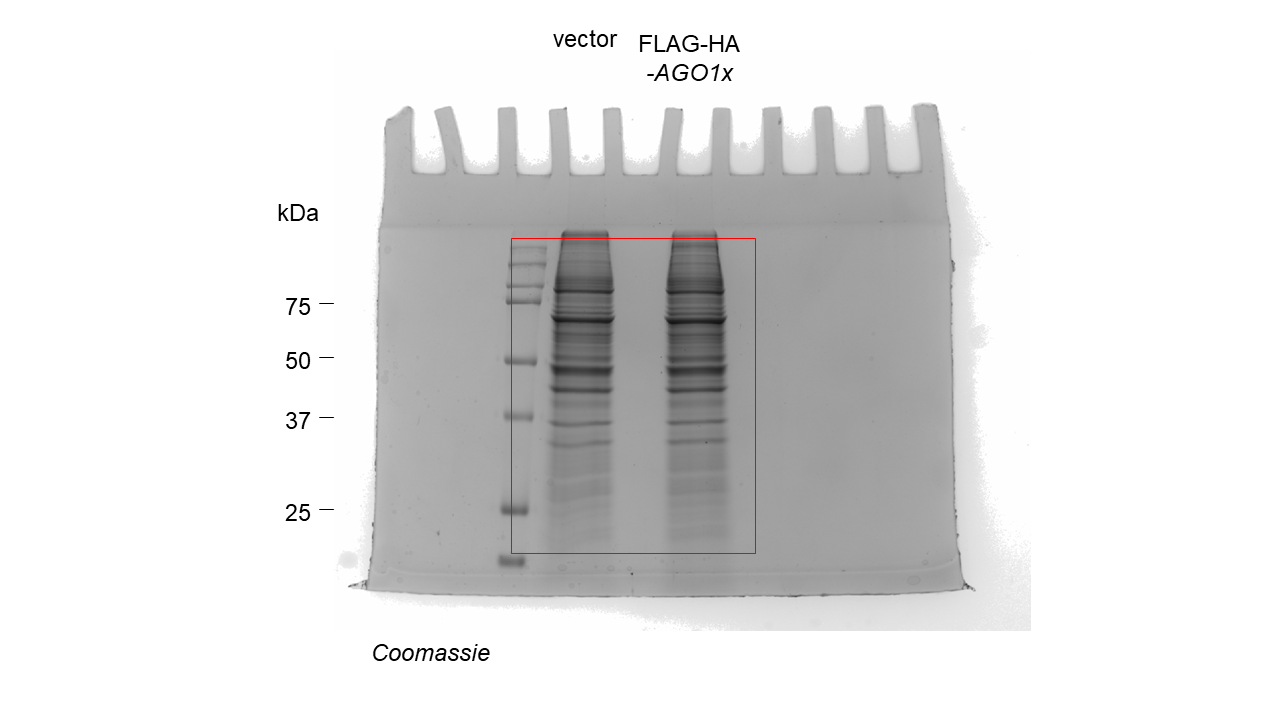

Supplement: Supplementary file 2 — Figure EV1 to EV5 Source Data [file 44319_2024_115_MOESM2_ESM.zip › EV Figures/Figure EV3/Figure EV3E/Coomassie.TIF]

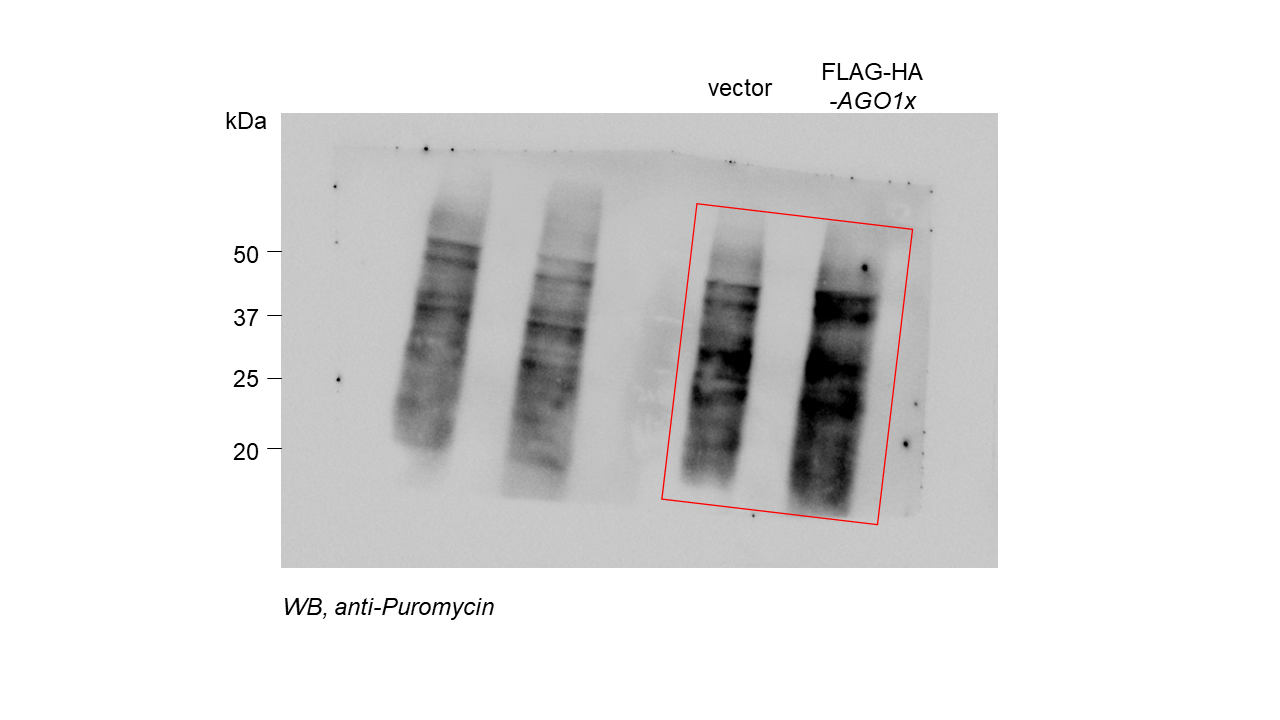

Supplement: Supplementary file 2 — Figure EV1 to EV5 Source Data [file 44319_2024_115_MOESM2_ESM.zip › EV Figures/Figure EV3/Figure EV3E/WB, anti- Puromycin.TIF]

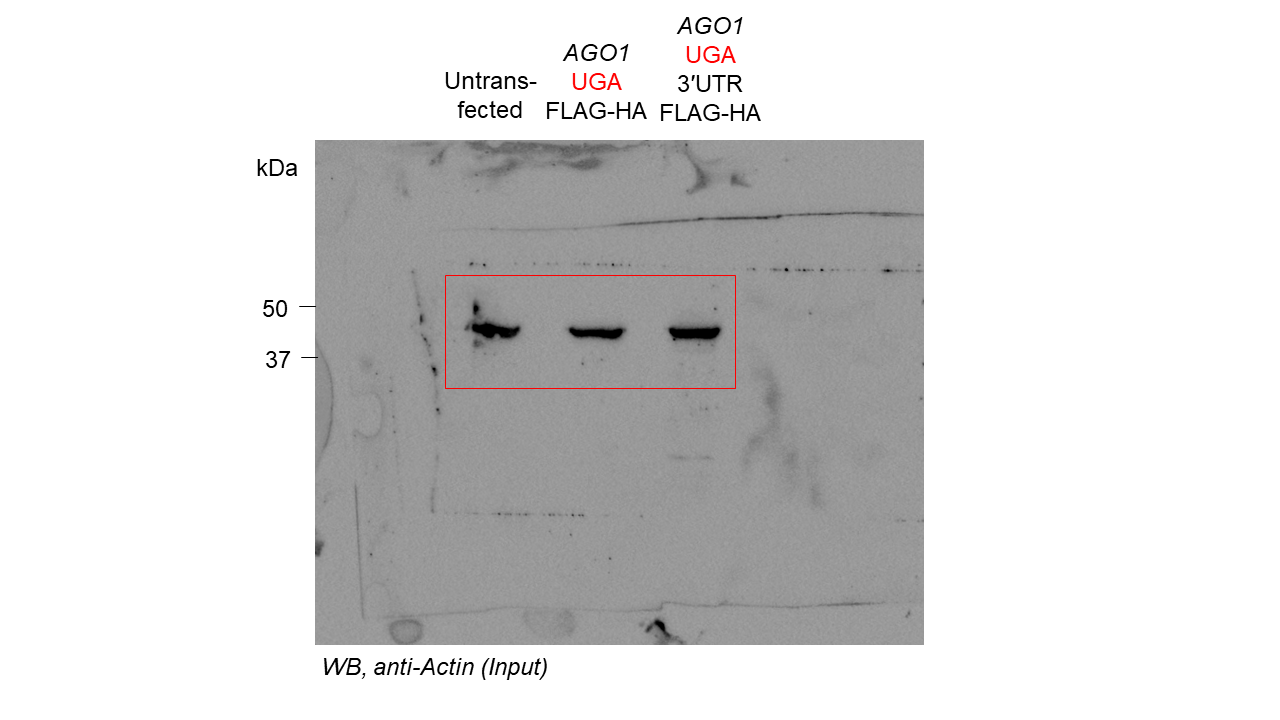

Supplement: Supplementary file 3 — Source Data Fig. 1 [file 44319_2024_115_MOESM3_ESM.zip › Figure 1/Figure 1C/WB, anti-Actin (left).TIF]

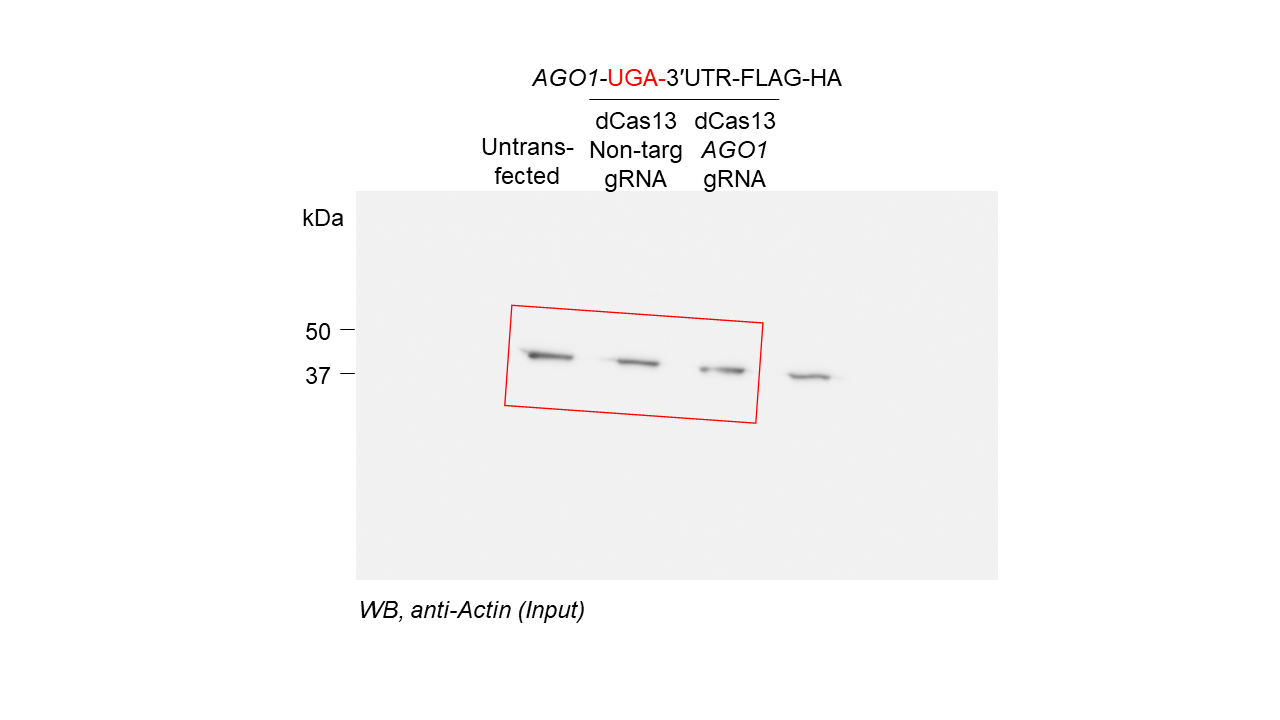

Supplement: Supplementary file 3 — Source Data Fig. 1 [file 44319_2024_115_MOESM3_ESM.zip › Figure 1/Figure 1C/WB, anti-Actin (right).TIF]

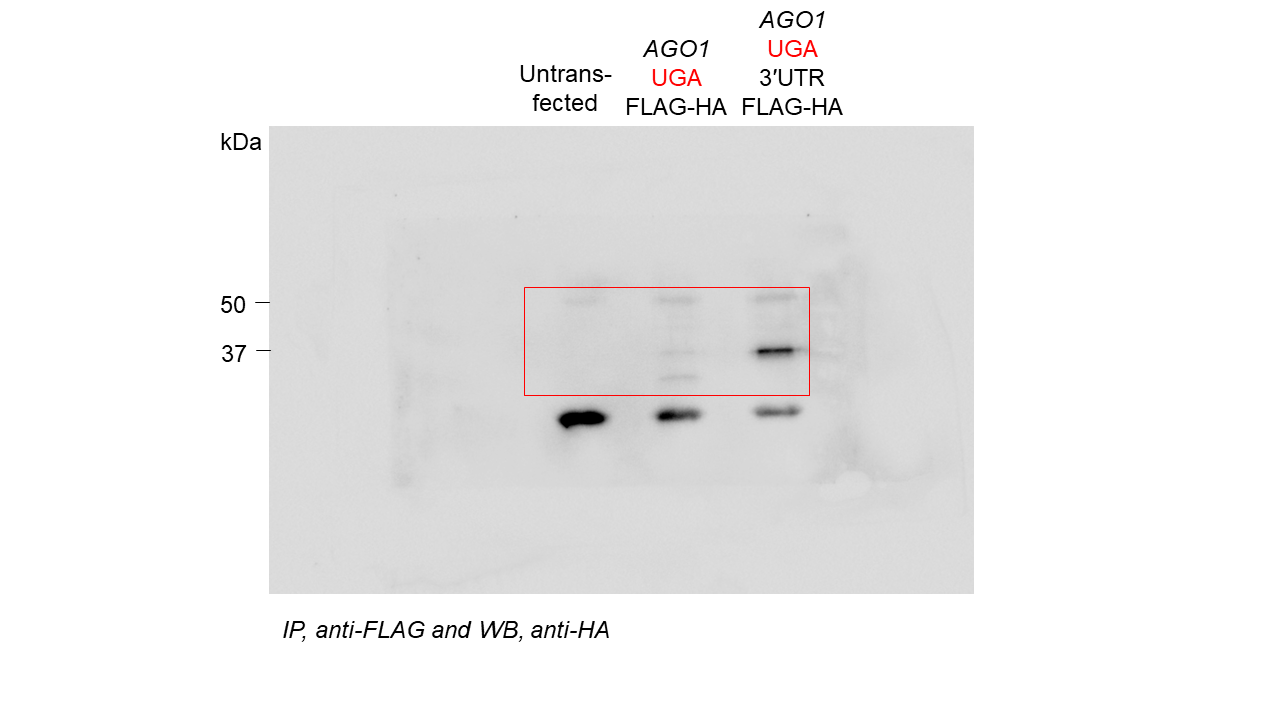

Supplement: Supplementary file 3 — Source Data Fig. 1 [file 44319_2024_115_MOESM3_ESM.zip › Figure 1/Figure 1C/WB, anti-HA (left).TIF]

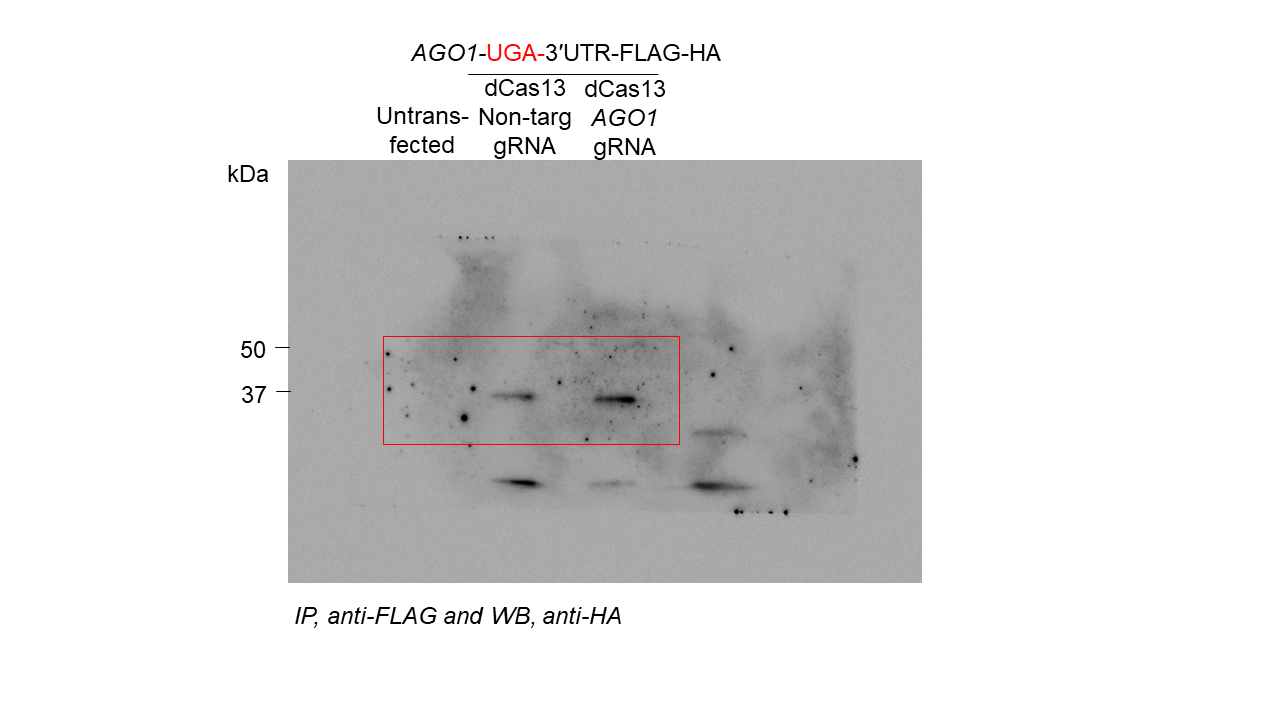

Supplement: Supplementary file 3 — Source Data Fig. 1 [file 44319_2024_115_MOESM3_ESM.zip › Figure 1/Figure 1C/WB, anti-HA (right).TIF]

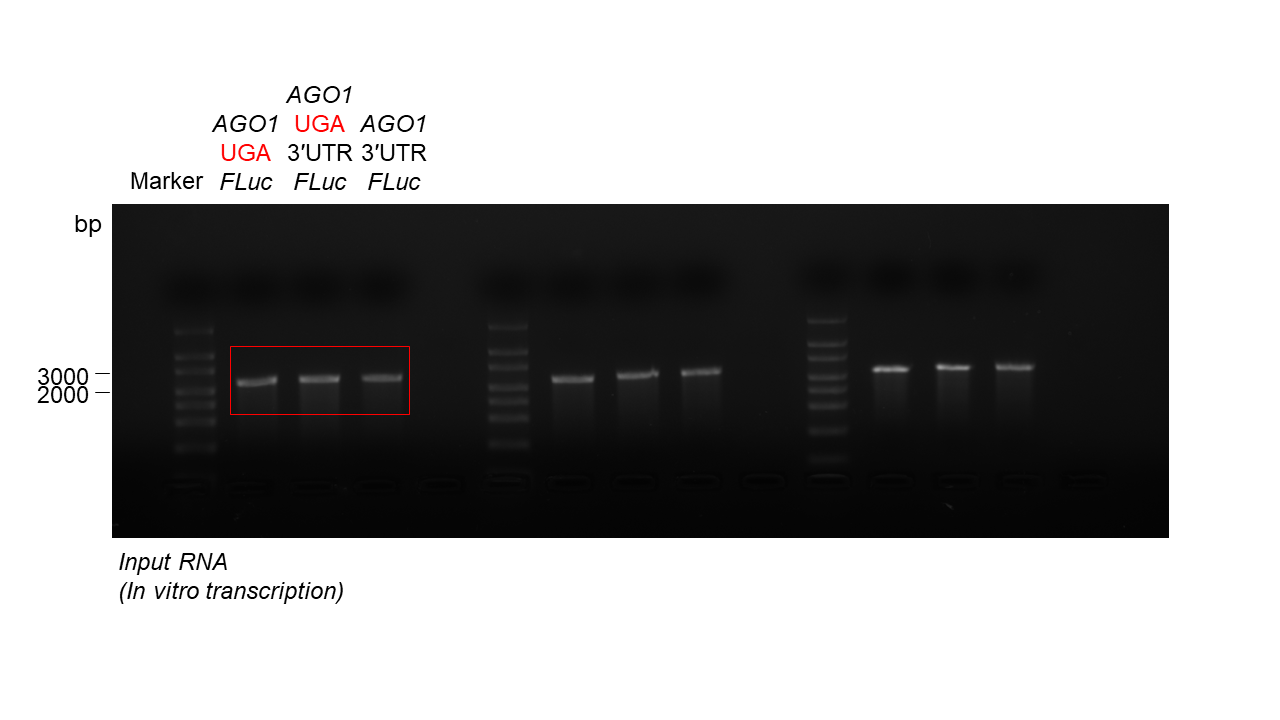

Supplement: Supplementary file 3 — Source Data Fig. 1 [file 44319_2024_115_MOESM3_ESM.zip › Figure 1/Figure 1E/Input RNA (In vitro transcription).TIF]

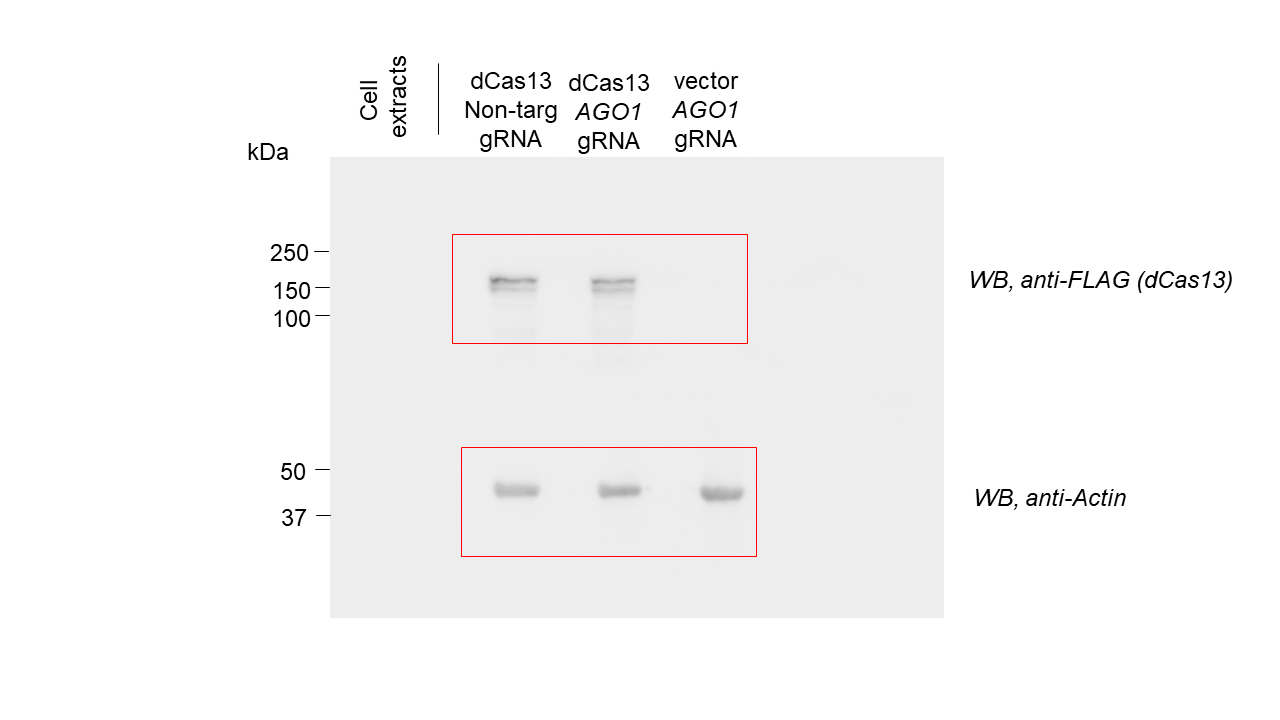

Supplement: Supplementary file 3 — Source Data Fig. 1 [file 44319_2024_115_MOESM3_ESM.zip › Figure 1/Figure 1F/WB, anti-FLAG(dCas13) and anti-Actin.TIF]

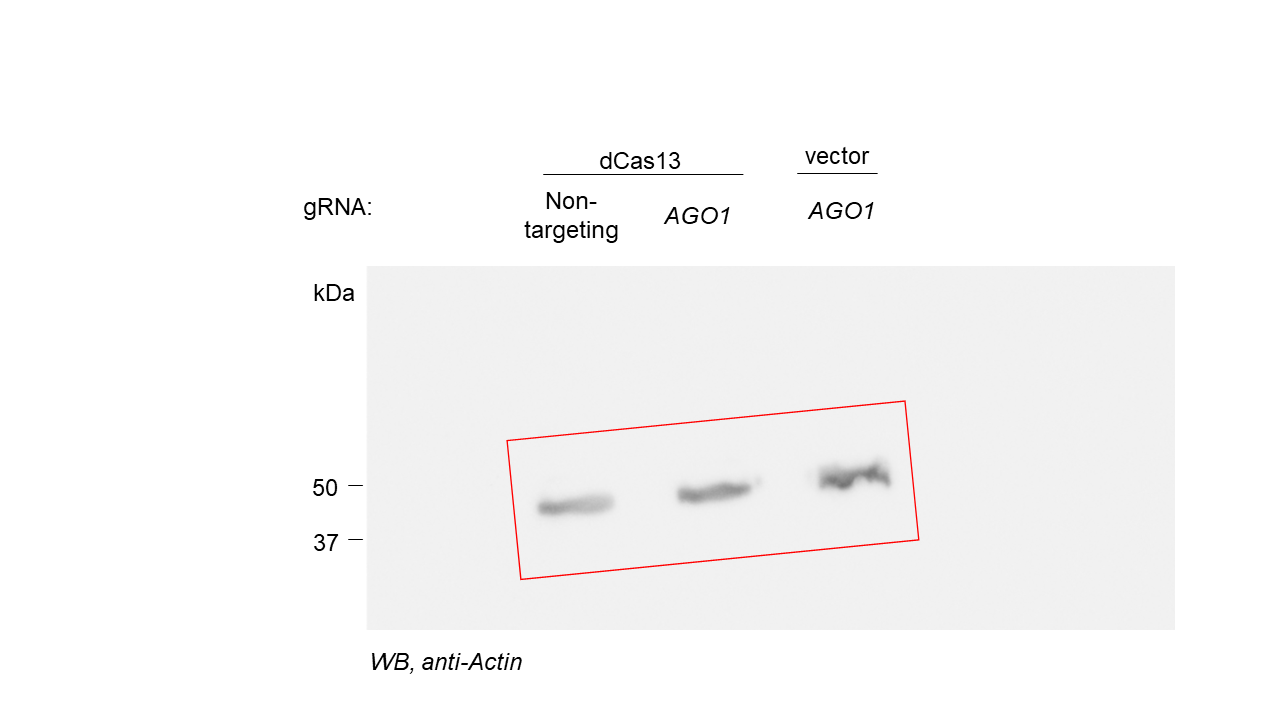

Supplement: Supplementary file 4 — Source Data Fig. 2 [file 44319_2024_115_MOESM4_ESM.zip › Figure 2/Figure 2A/WB, anti-Actin (Left).TIF]

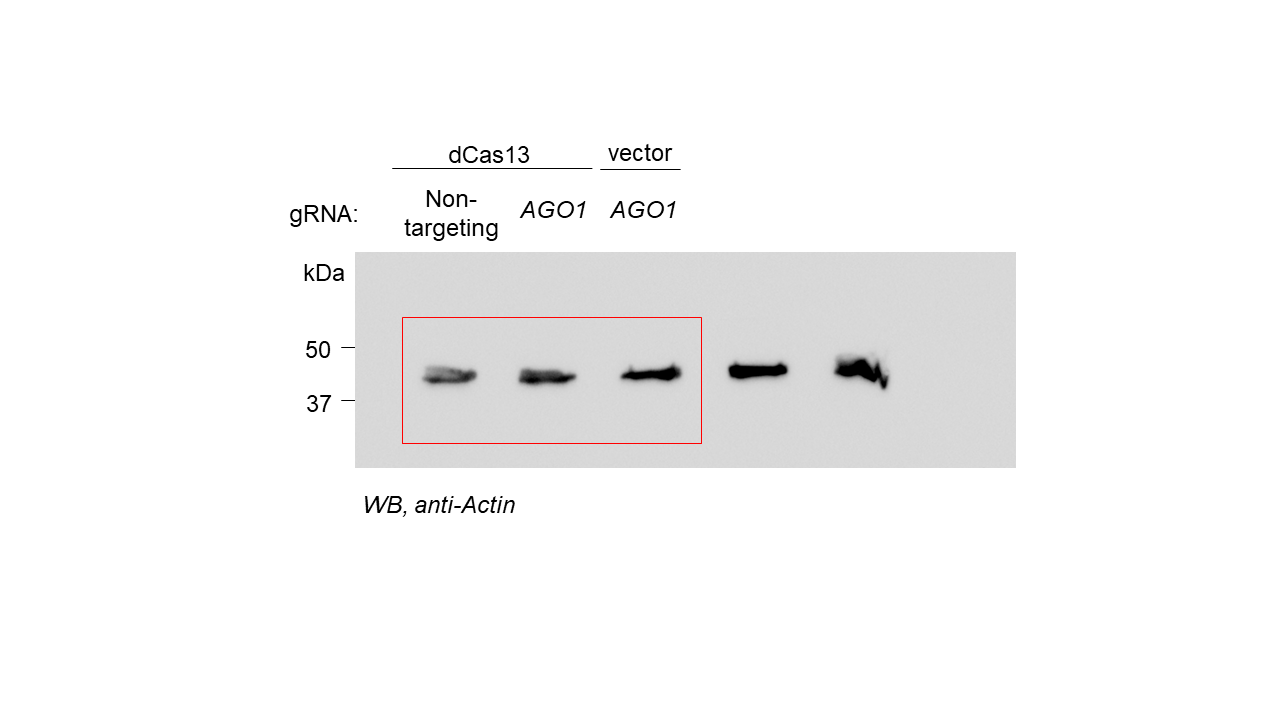

Supplement: Supplementary file 4 — Source Data Fig. 2 [file 44319_2024_115_MOESM4_ESM.zip › Figure 2/Figure 2A/WB, anti-Actin (Right).TIF]

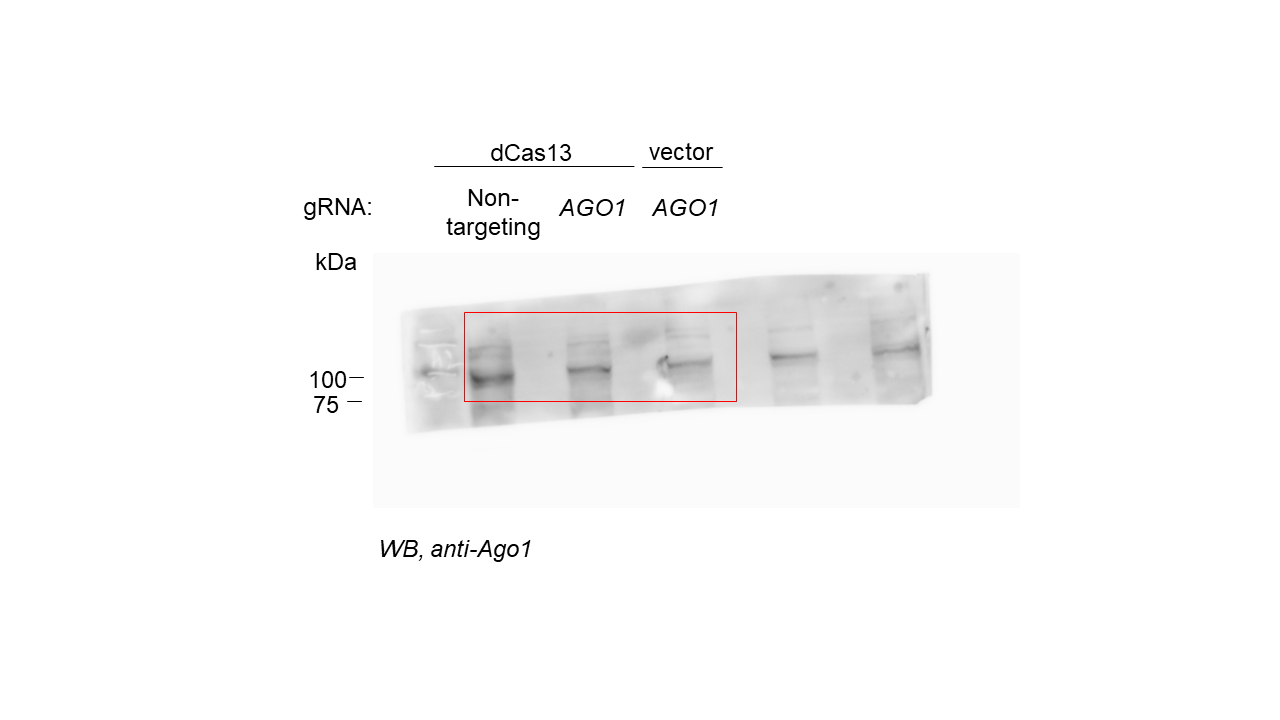

Supplement: Supplementary file 4 — Source Data Fig. 2 [file 44319_2024_115_MOESM4_ESM.zip › Figure 2/Figure 2A/WB, anti-Ago1.TIF]

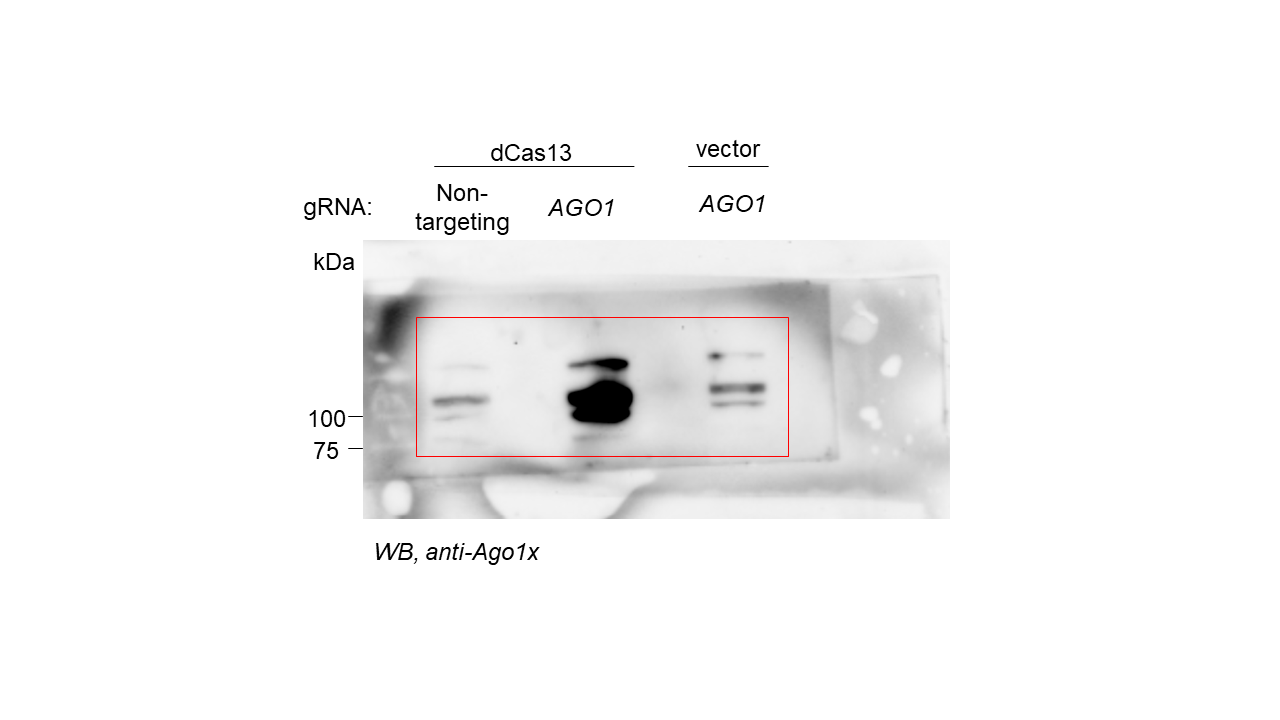

Supplement: Supplementary file 4 — Source Data Fig. 2 [file 44319_2024_115_MOESM4_ESM.zip › Figure 2/Figure 2A/WB, anti-Ago1x.TIF]

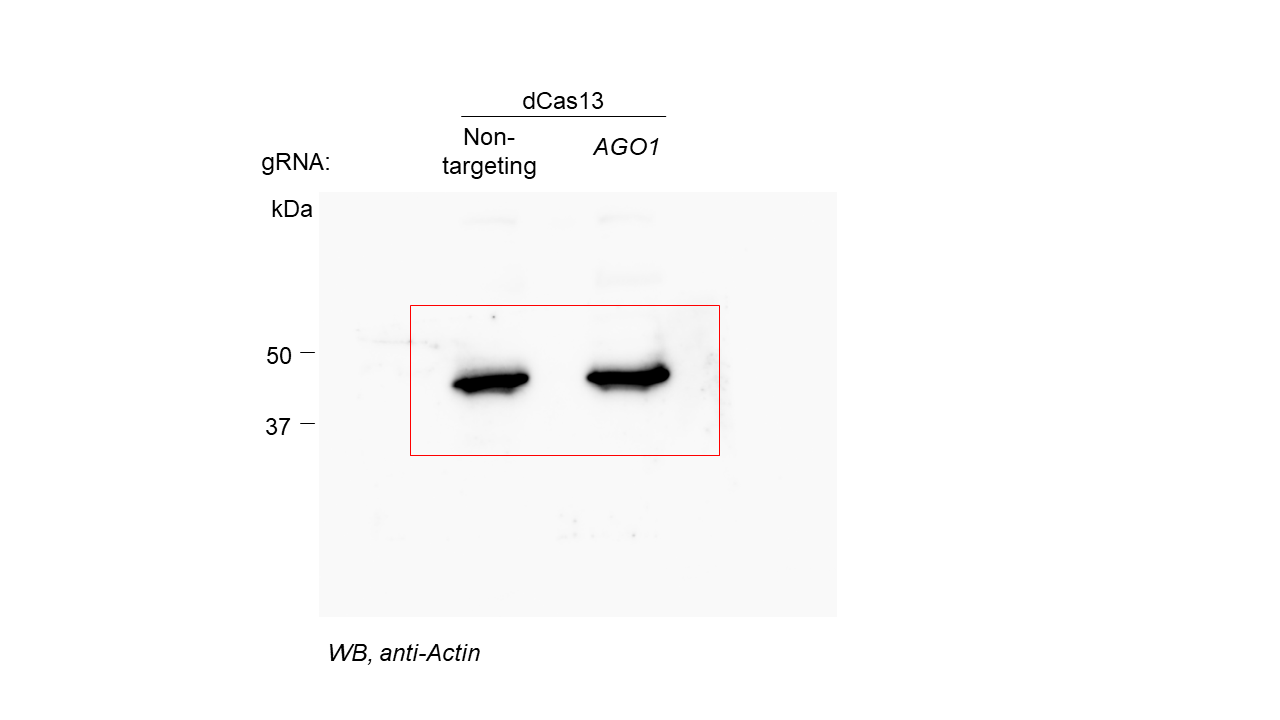

Supplement: Supplementary file 4 — Source Data Fig. 2 [file 44319_2024_115_MOESM4_ESM.zip › Figure 2/Figure 2C/WB, anti-Actin.TIF]

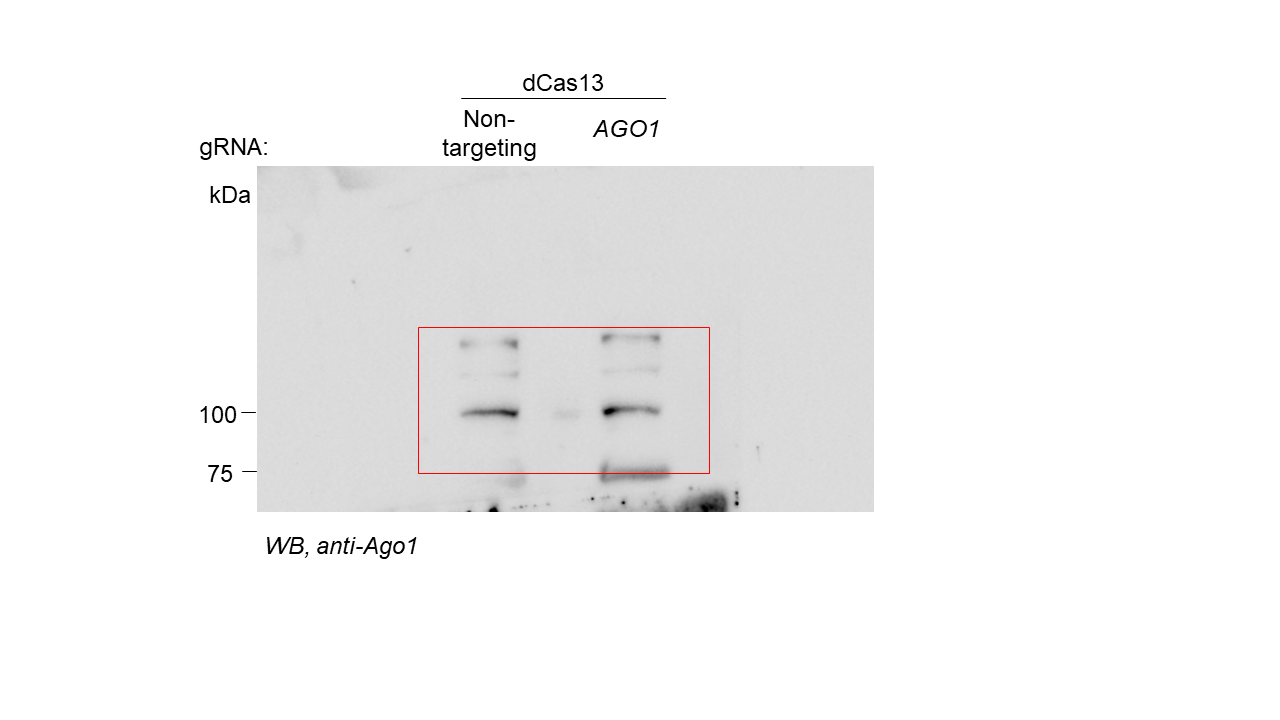

Supplement: Supplementary file 4 — Source Data Fig. 2 [file 44319_2024_115_MOESM4_ESM.zip › Figure 2/Figure 2C/WB, anti-Ago1.TIF]

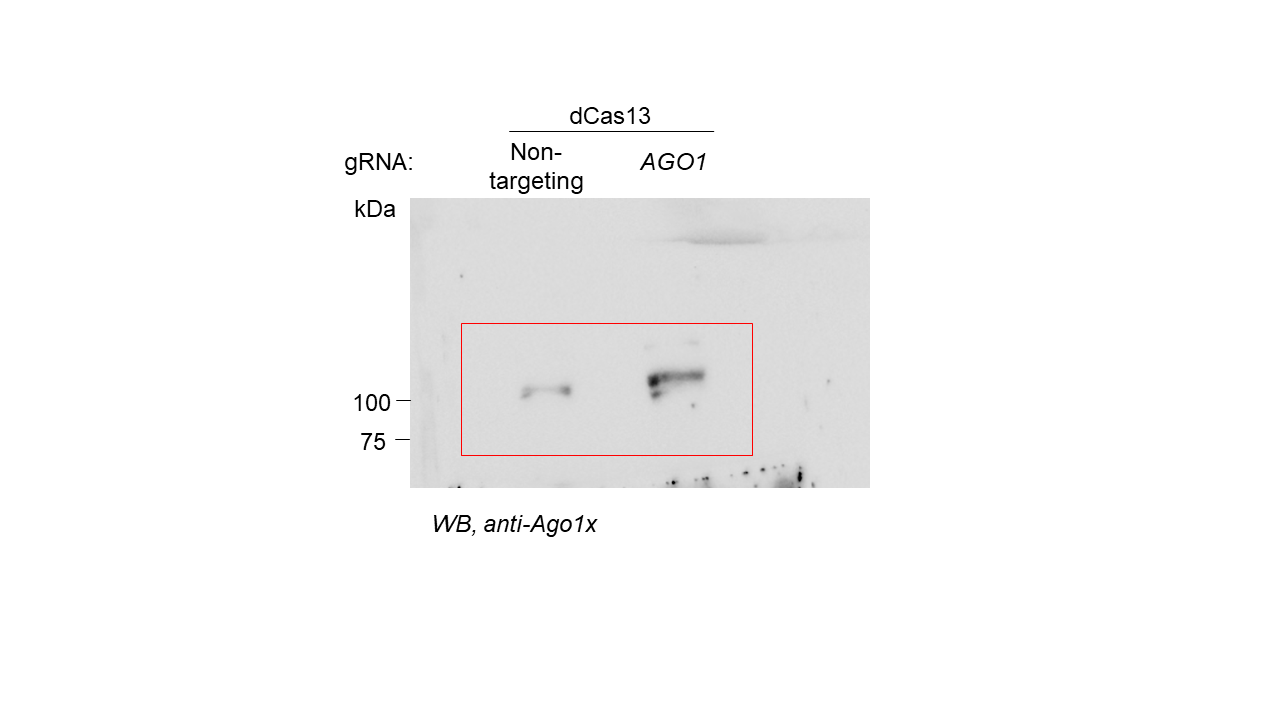

Supplement: Supplementary file 4 — Source Data Fig. 2 [file 44319_2024_115_MOESM4_ESM.zip › Figure 2/Figure 2C/WB, anti-Ago1x.TIF]

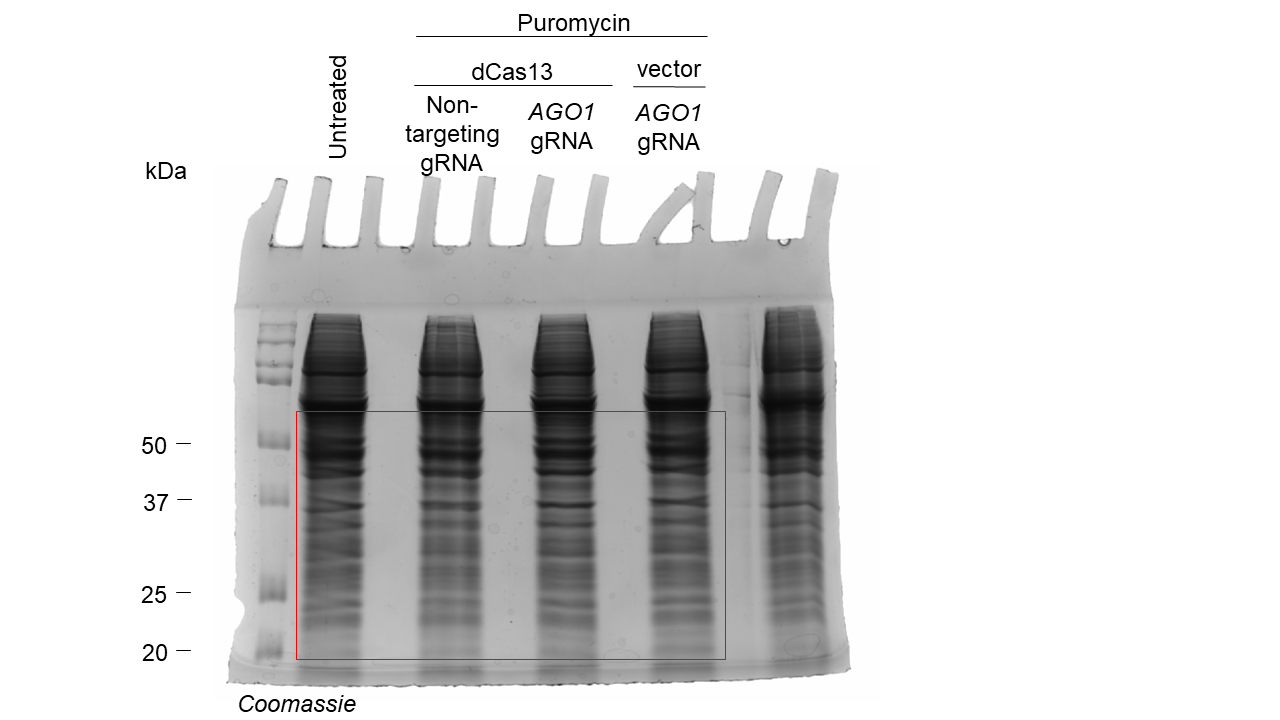

Supplement: Supplementary file 5 — Source Data Fig. 3 [file 44319_2024_115_MOESM5_ESM.zip › Figure 3/Figure 3A/Coomassie.TIF]

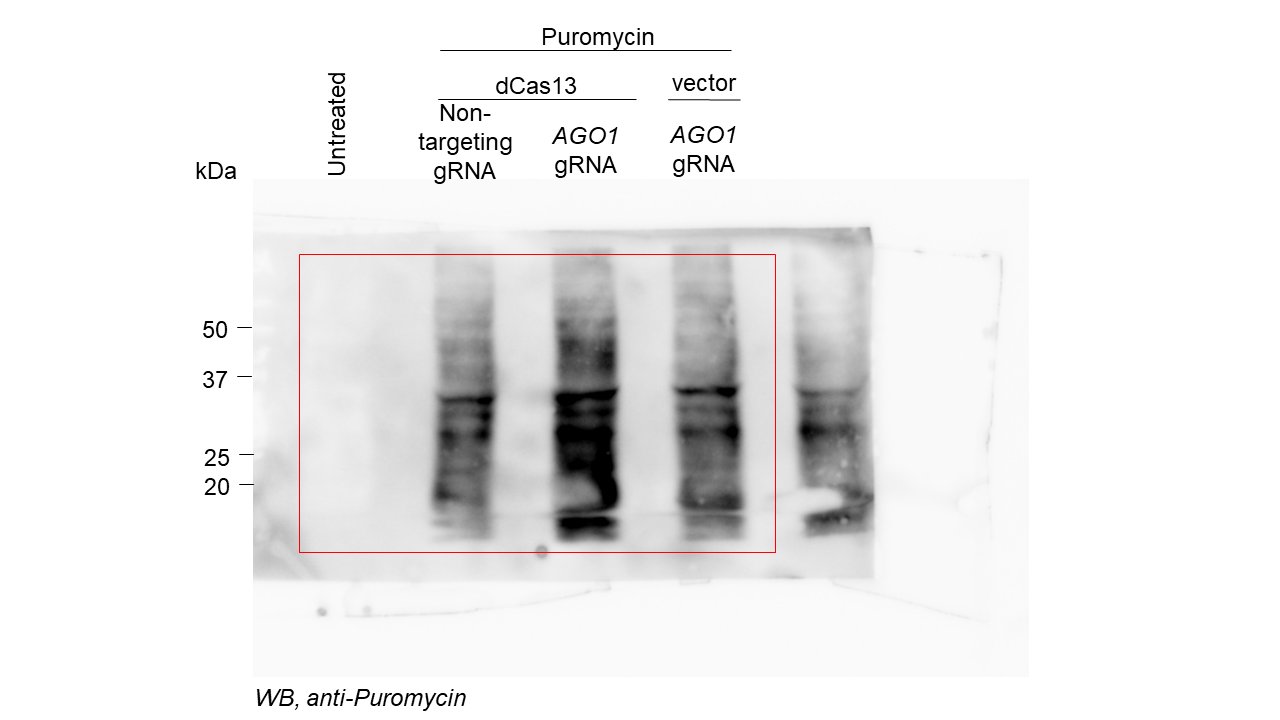

Supplement: Supplementary file 5 — Source Data Fig. 3 [file 44319_2024_115_MOESM5_ESM.zip › Figure 3/Figure 3A/WB, anti-Puromycin.TIF]

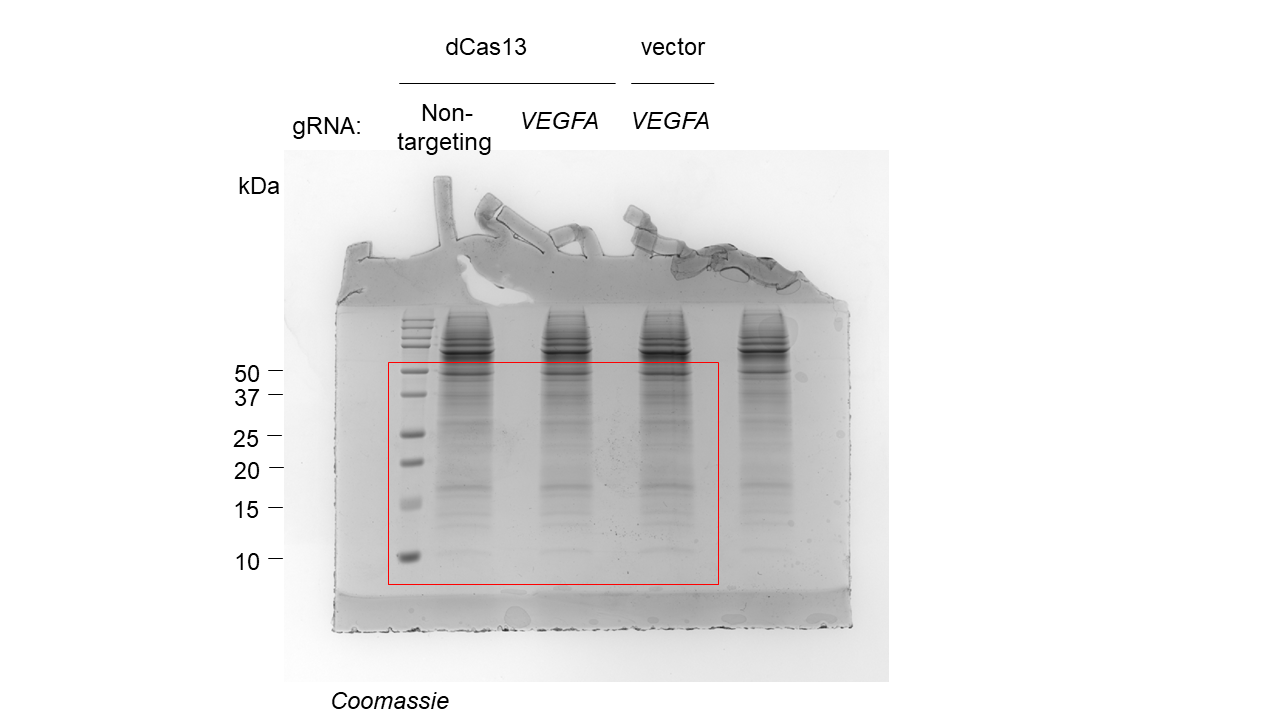

Supplement: Supplementary file 7 — Source Data Fig. 5 [file 44319_2024_115_MOESM7_ESM.zip › Figure 5/Figure 5B/Coomassie.TIF]

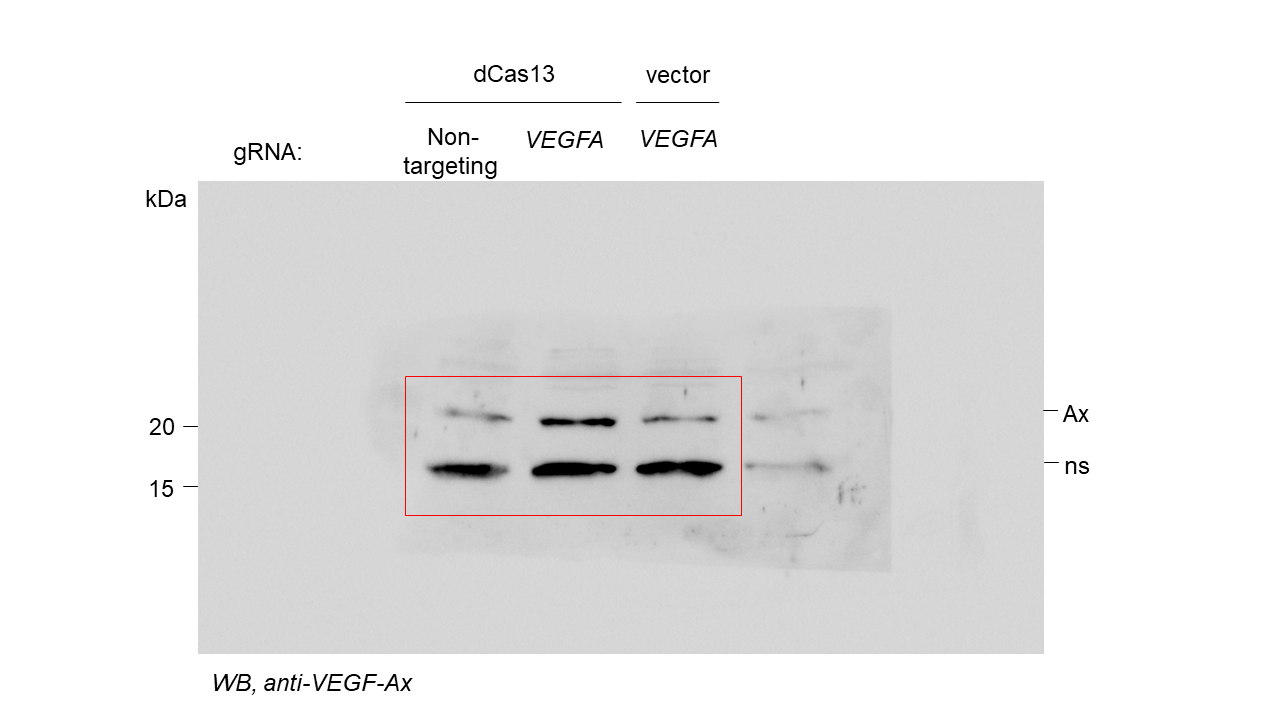

Supplement: Supplementary file 7 — Source Data Fig. 5 [file 44319_2024_115_MOESM7_ESM.zip › Figure 5/Figure 5B/WB, anti-VEGF-Ax.TIF]

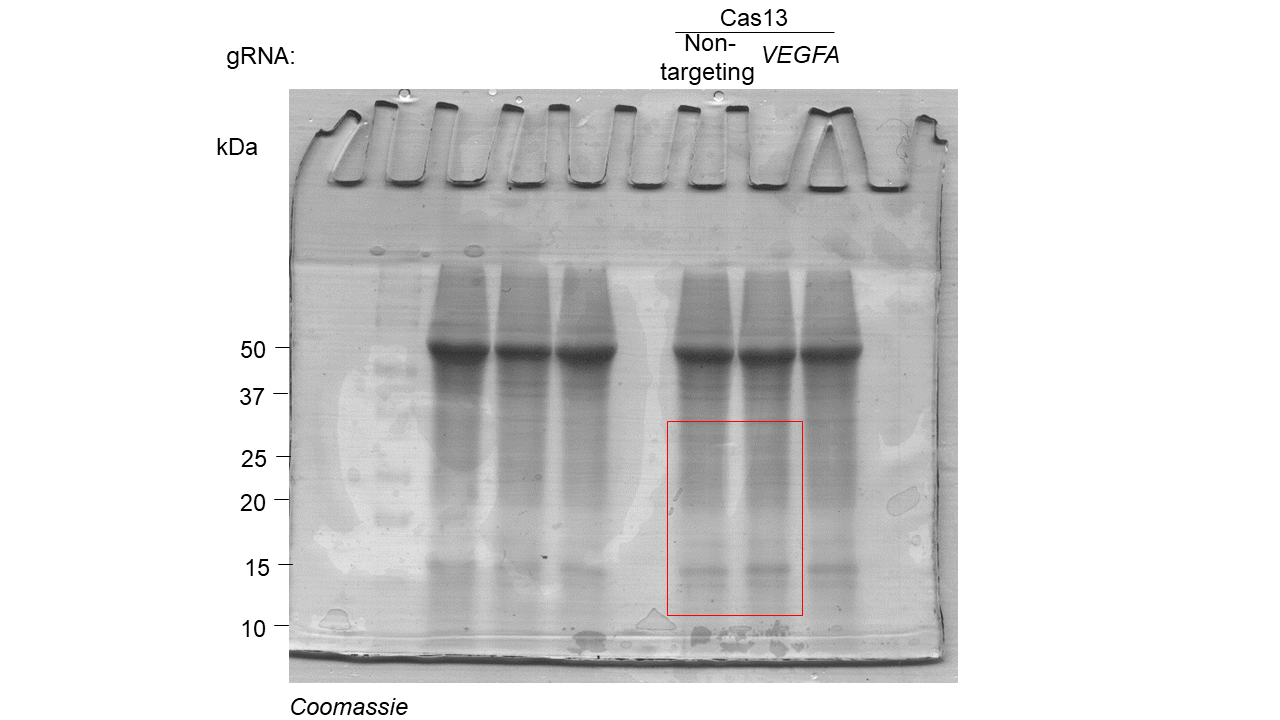

Supplement: Supplementary file 7 — Source Data Fig. 5 [file 44319_2024_115_MOESM7_ESM.zip › Figure 5/Figure 5D/Coomassie.TIF]

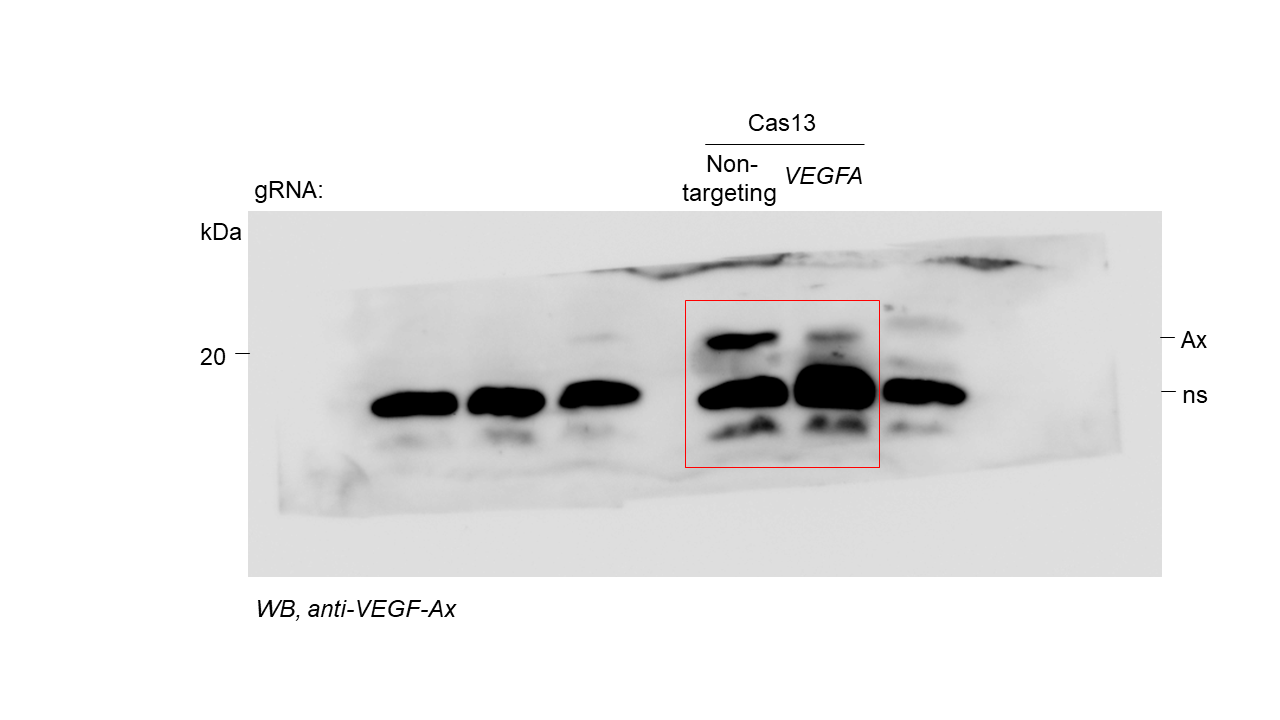

Supplement: Supplementary file 7 — Source Data Fig. 5 [file 44319_2024_115_MOESM7_ESM.zip › Figure 5/Figure 5D/WB, anti-VEGF-Ax.TIF]

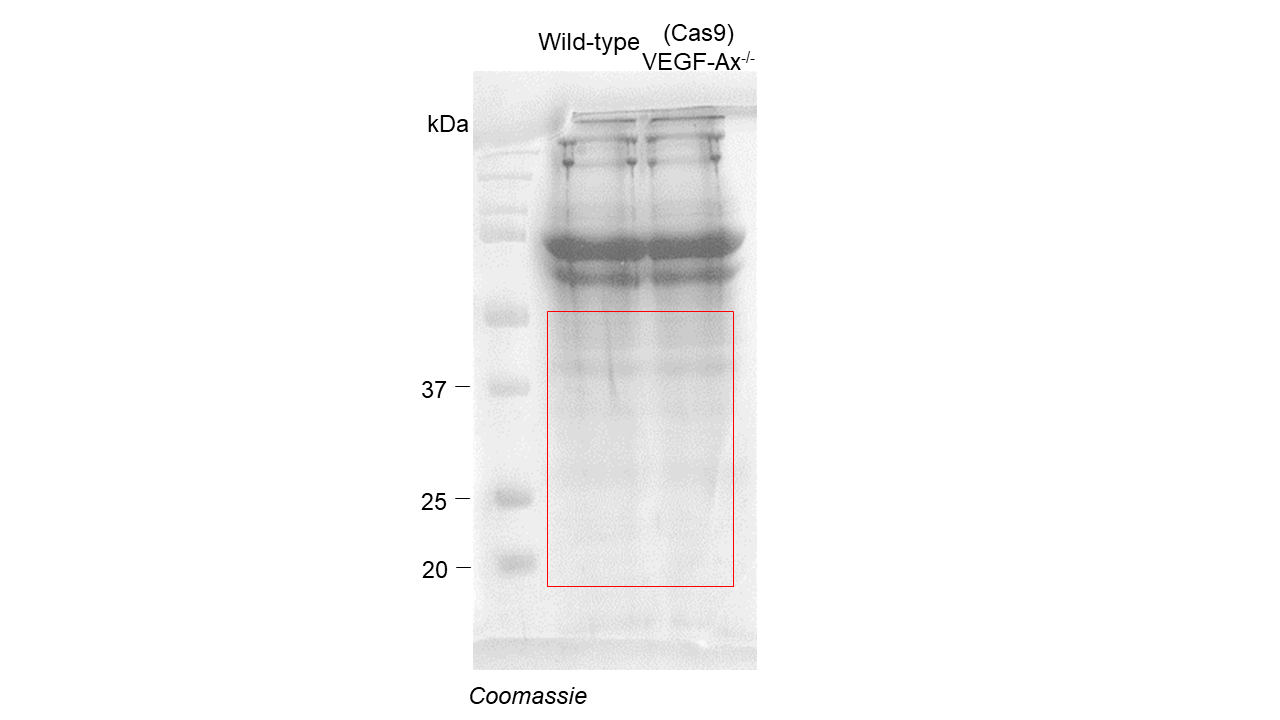

Supplement: Supplementary file 7 — Source Data Fig. 5 [file 44319_2024_115_MOESM7_ESM.zip › Figure 5/Figure 5E/Coomassie.TIF]

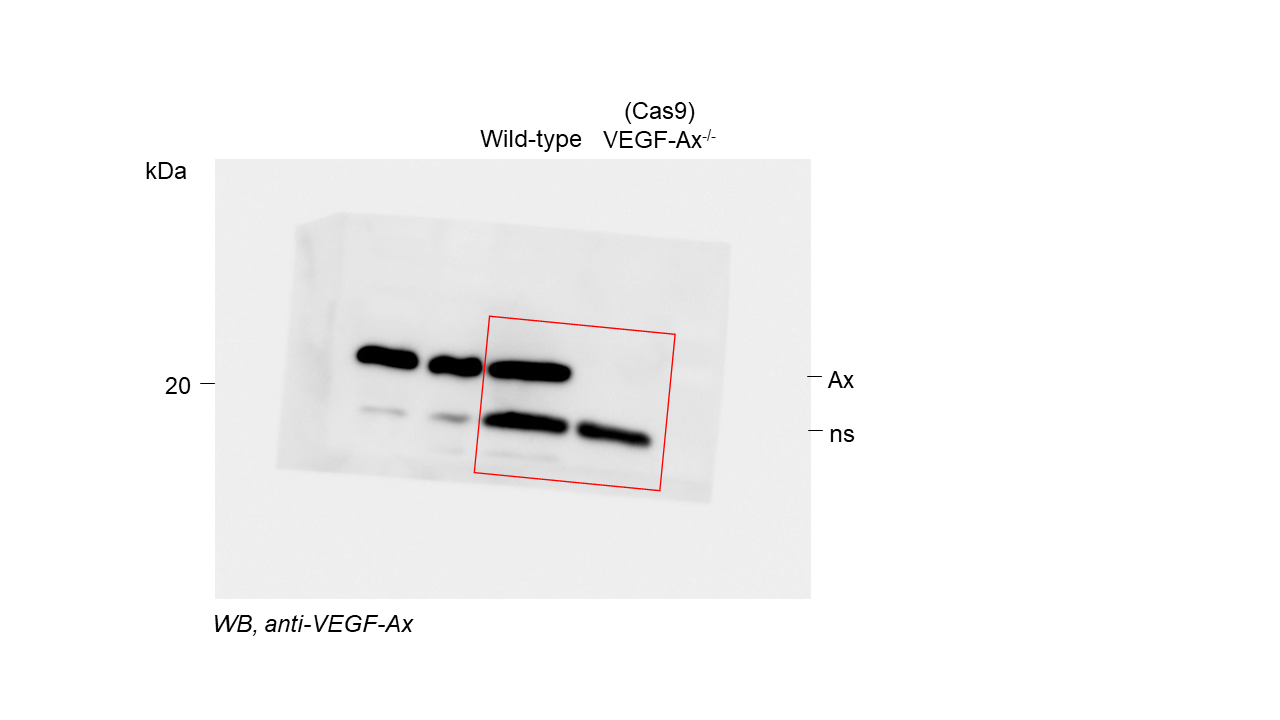

Supplement: Supplementary file 7 — Source Data Fig. 5 [file 44319_2024_115_MOESM7_ESM.zip › Figure 5/Figure 5E/WB, anti-VEGF-Ax.TIF]

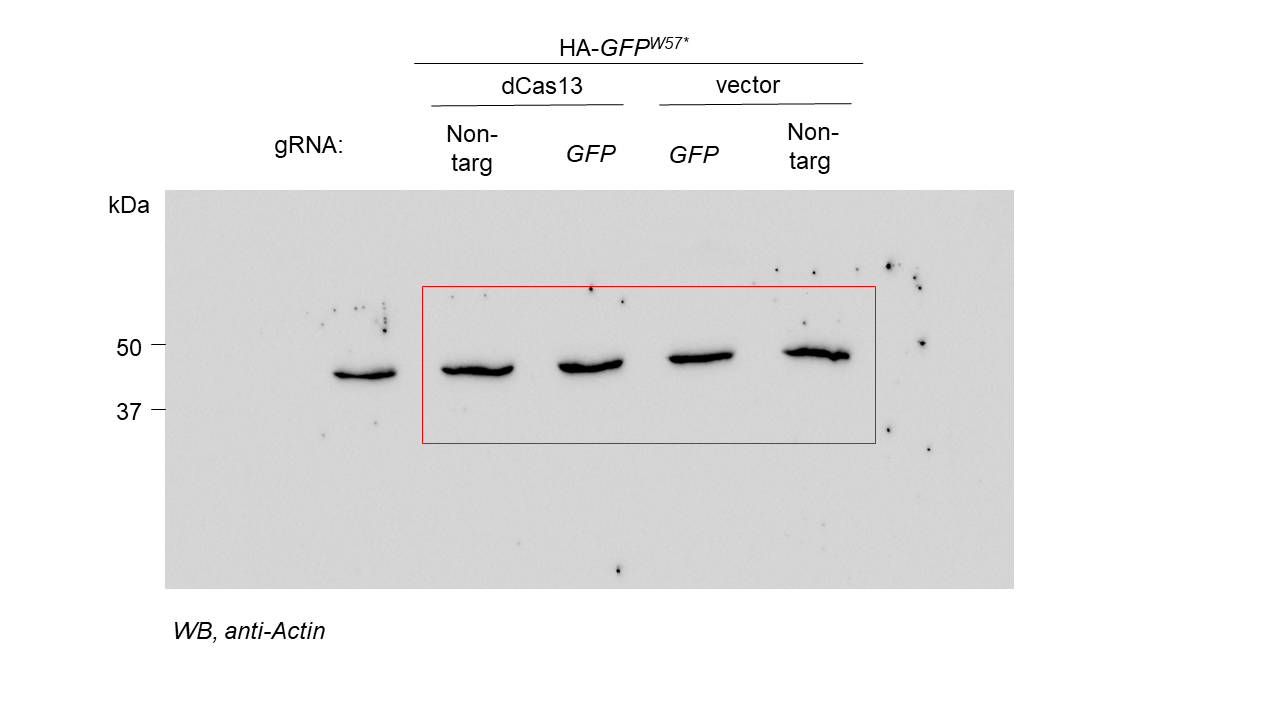

Supplement: Supplementary file 8 — Source Data Fig. 6 [file 44319_2024_115_MOESM8_ESM.zip › Figure 6/Figure 6A/WB, anti-Actin.TIF]

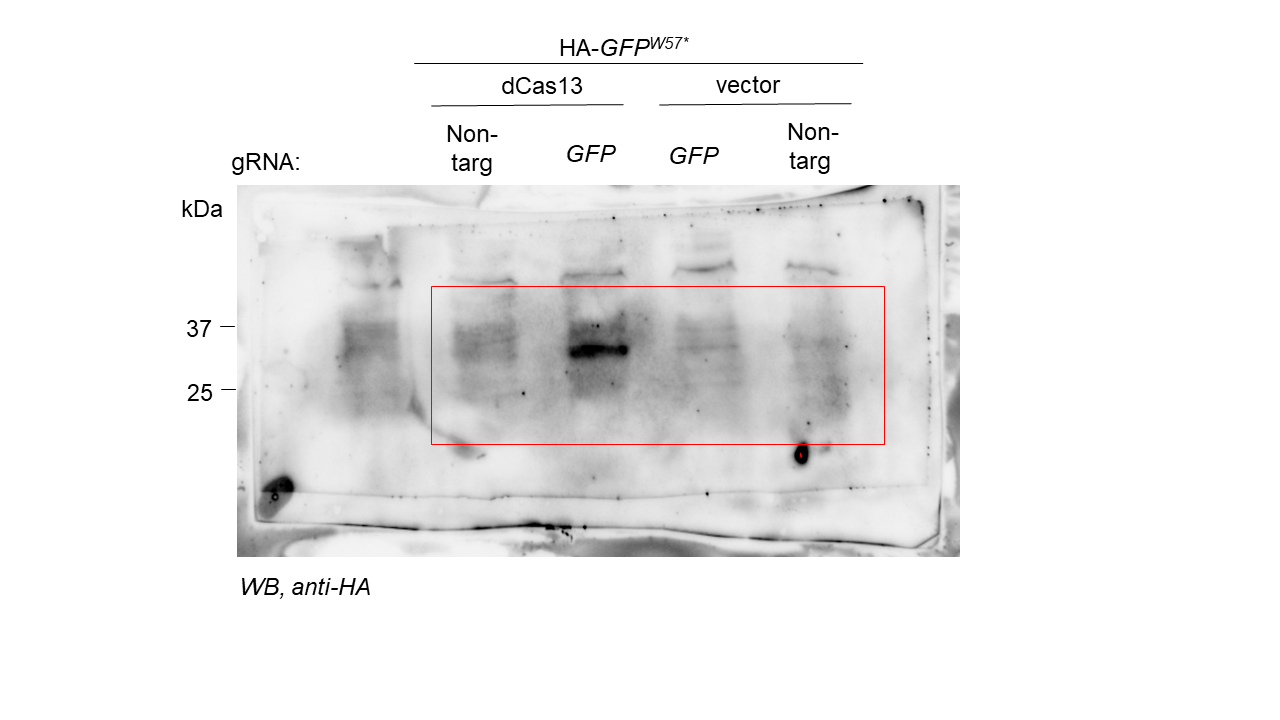

Supplement: Supplementary file 8 — Source Data Fig. 6 [file 44319_2024_115_MOESM8_ESM.zip › Figure 6/Figure 6A/WB, anti-HA.TIF]

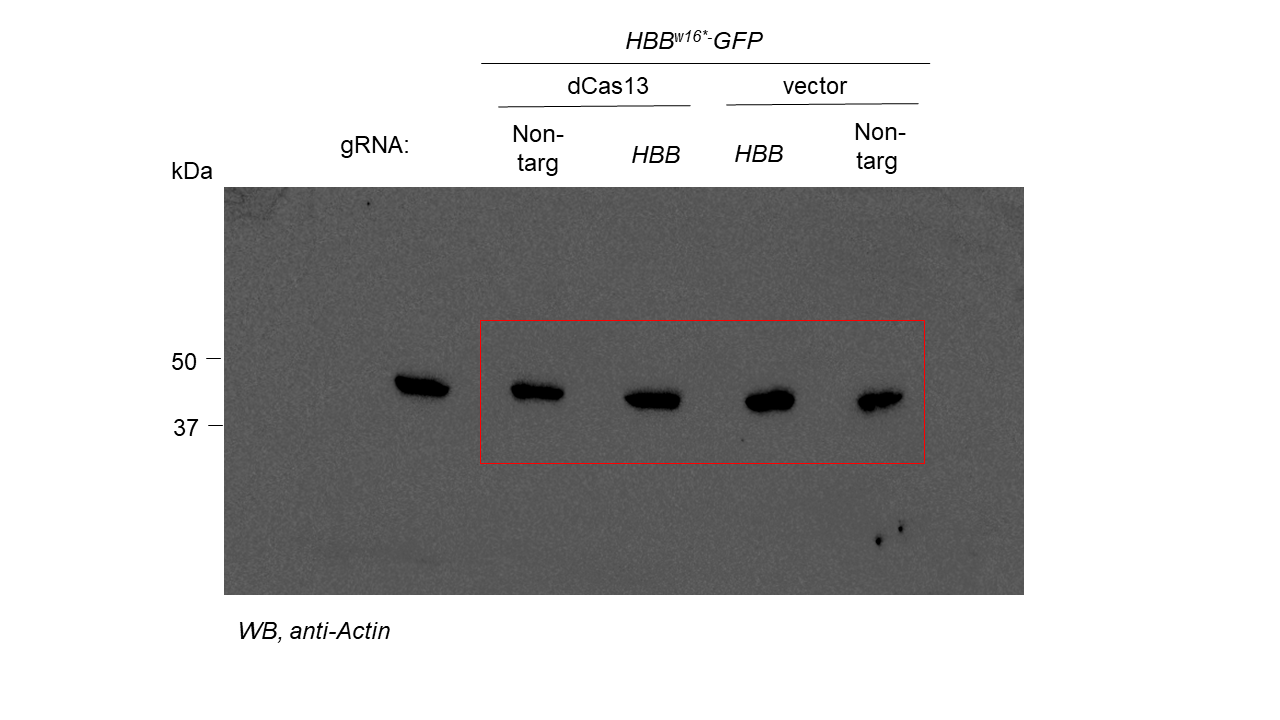

Supplement: Supplementary file 8 — Source Data Fig. 6 [file 44319_2024_115_MOESM8_ESM.zip › Figure 6/Figure 6B/WB, anti-Actin.TIF]

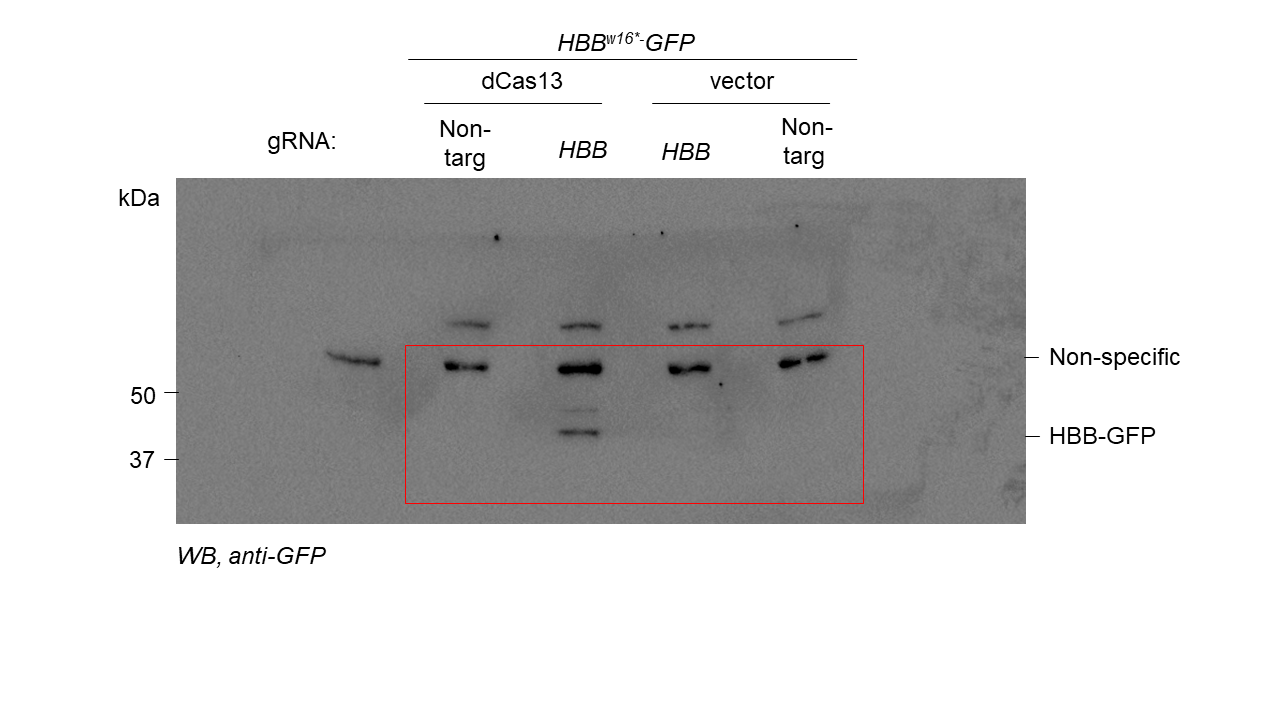

Supplement: Supplementary file 8 — Source Data Fig. 6 [file 44319_2024_115_MOESM8_ESM.zip › Figure 6/Figure 6B/WB, anti-GFP.TIF]

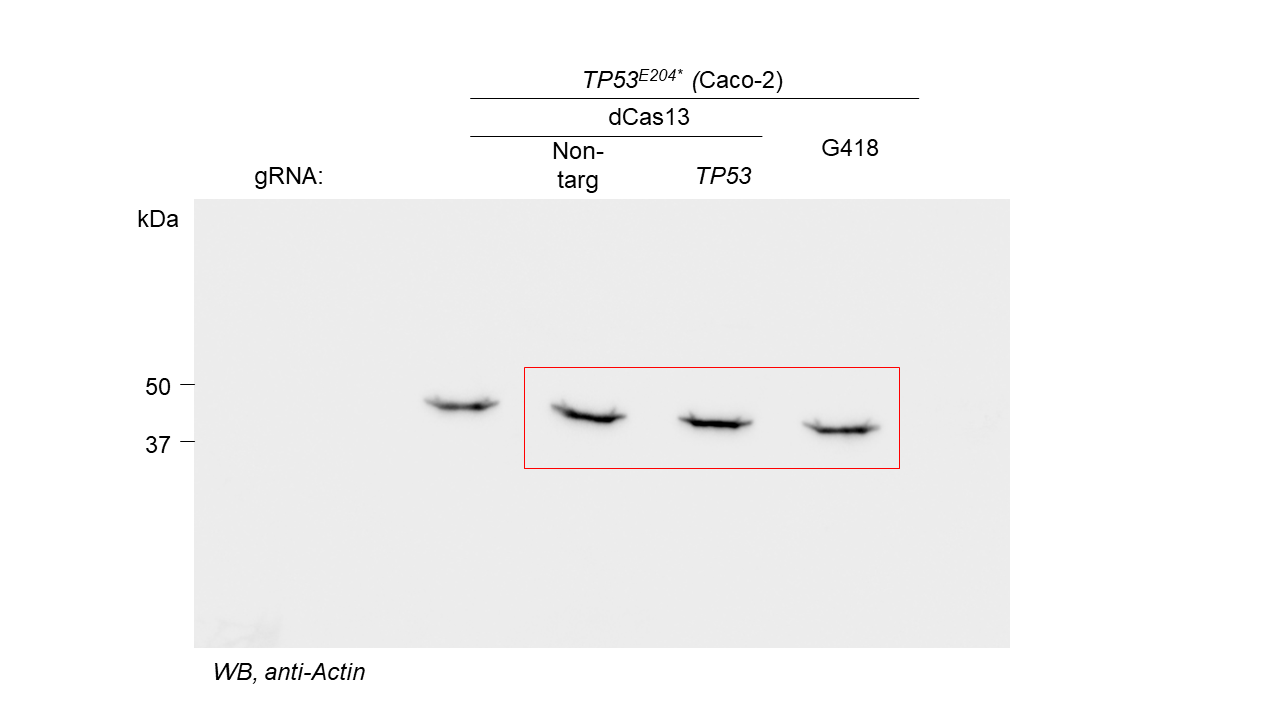

Supplement: Supplementary file 8 — Source Data Fig. 6 [file 44319_2024_115_MOESM8_ESM.zip › Figure 6/Figure 6C/WB, anti-Actin.TIF]

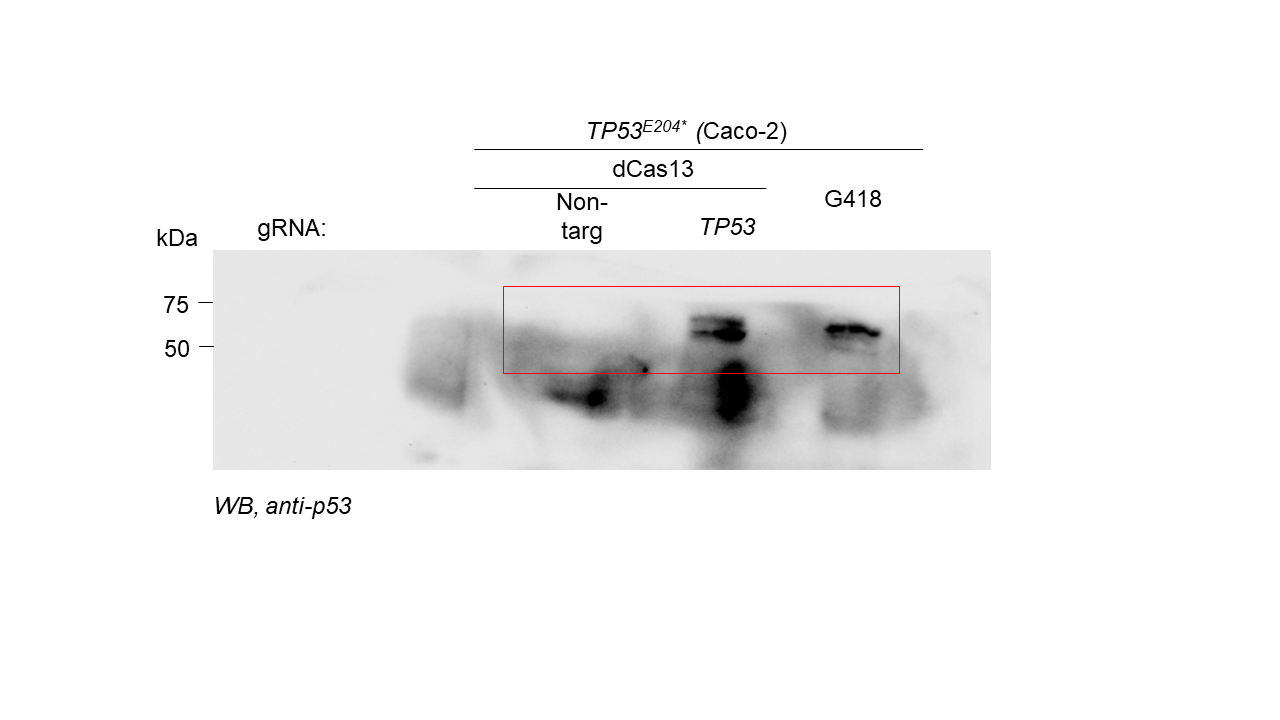

Supplement: Supplementary file 8 — Source Data Fig. 6 [file 44319_2024_115_MOESM8_ESM.zip › Figure 6/Figure 6C/WB, anti-p53.TIF]

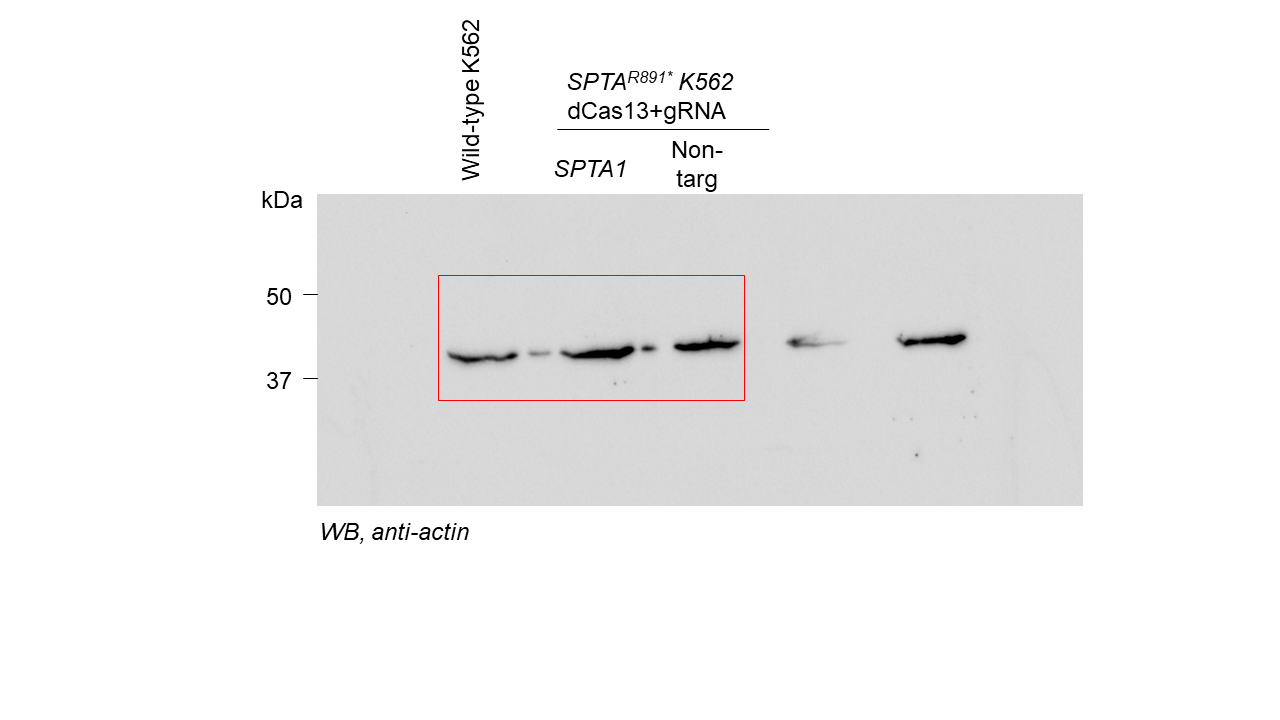

Supplement: Supplementary file 8 — Source Data Fig. 6 [file 44319_2024_115_MOESM8_ESM.zip › Figure 6/Figure 6D/WB, anti-Actin (Left).TIF]

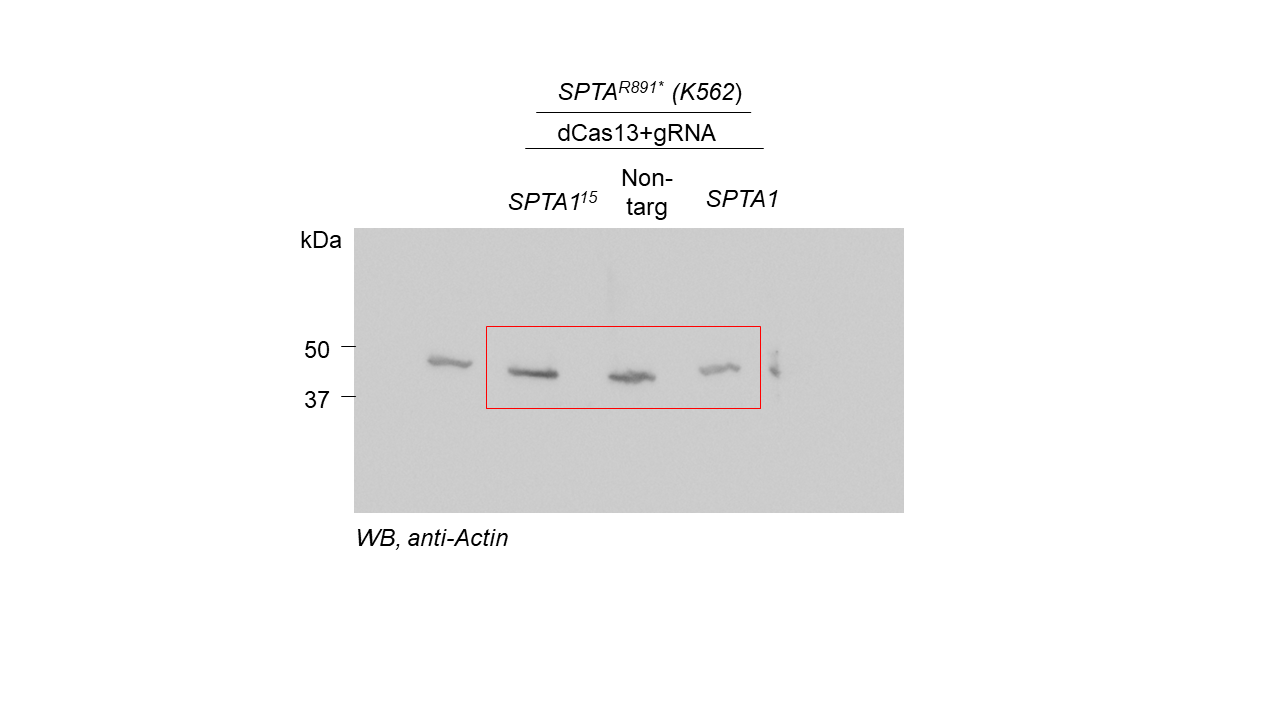

Supplement: Supplementary file 8 — Source Data Fig. 6 [file 44319_2024_115_MOESM8_ESM.zip › Figure 6/Figure 6D/WB, anti-Actin (Right).TIF]

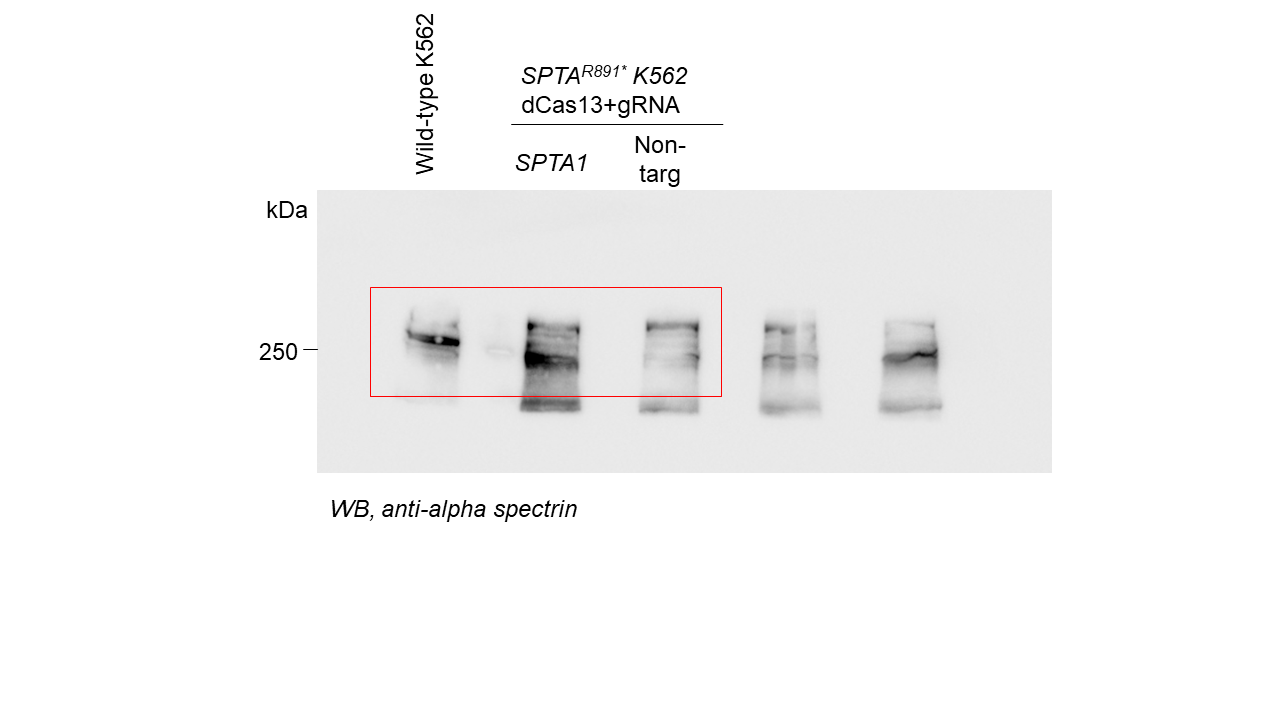

Supplement: Supplementary file 8 — Source Data Fig. 6 [file 44319_2024_115_MOESM8_ESM.zip › Figure 6/Figure 6D/WB, anti-alpha spectrin (Left).TIF]

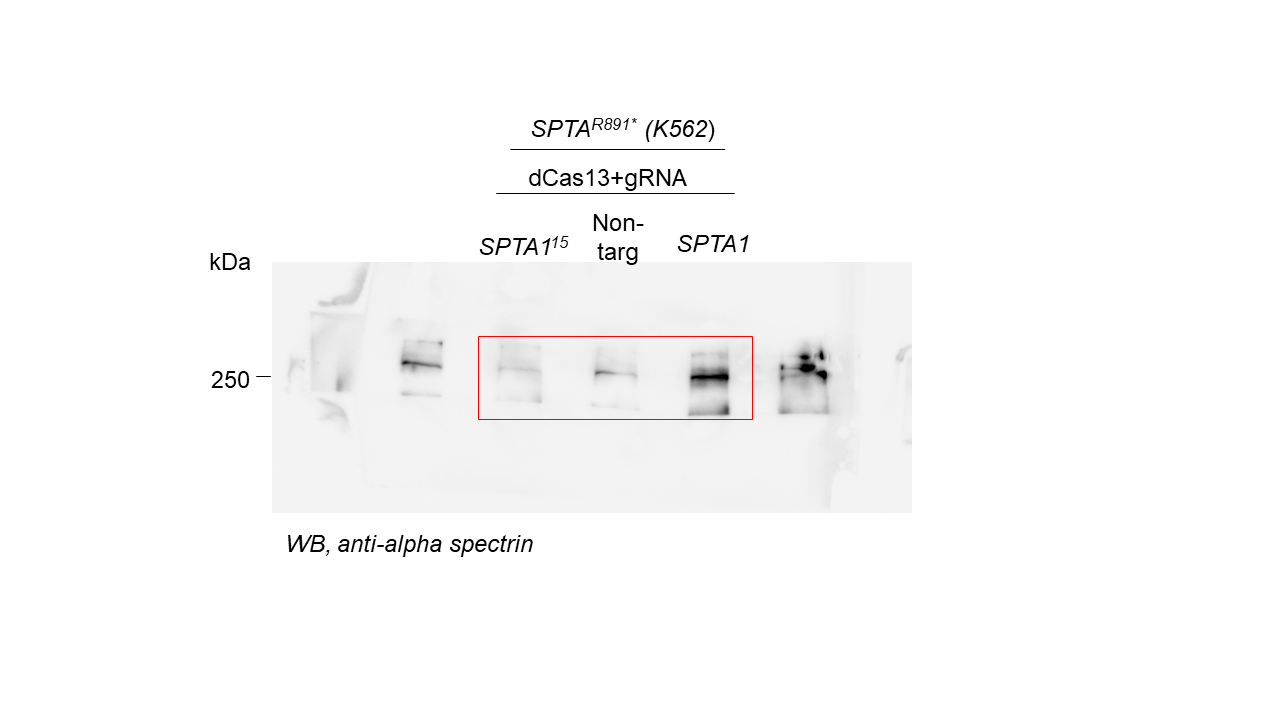

Supplement: Supplementary file 8 — Source Data Fig. 6 [file 44319_2024_115_MOESM8_ESM.zip › Figure 6/Figure 6D/WB, anti-alpha spectrin (Right).TIF]
