# Supplementary material for: Transcript-specific induction of stop codon readthrough using a CRISPR-dCas13 system
Source: EMBO Rep. 2024 Mar 18;25(4):26. doi: 10.1038/s44319-024-00115-8 (PMC11015002; doi:10.1038/s44319-024-00115-8)
Supplement: Supplementary file 10 — Expanded View Figures [file 44319_2024_115_MOESM10_ESM.pdf]

Expanded View Figures

Figure EV1. (**A**) RT-PCR analysis showing the expression of Cas13 and dCas13 (*L. wadei*) in transfected HEK293 cells. Following primers were used (5′ to 3′): AGAACAACAAGGGCGAAGAGAAAT and CTTGCCTTCCAGTTCCAGGT. (**B**) dCas13 interacts with its target mRNA, *AGO1*, mediated by a specific gRNA. Constructs expressing dPguCas13b-3xFLAG along with gRNAs were transfected in HEK293 cells. Cell lysates were subjected to immunoprecipitation followed by RNA isolation and qRT-PCR to detect the enrichment of *AGO1* mRNA. Immunoprecipitates were also used for western blotting. Data information: Graph, mean ± SD, $N = 3$ experiments. (**C**) Schematics of constructs used in western blotting-based (FLAG-HA tag) and luminescence-based (firefly luciferase) SCR assays described in Fig. 1. (**D**) Western blotting-based SCR assay. The indicated plasmid constructs were transfected in HEK293 cells. After 48 h, they were subjected to immunoprecipitation followed by western blotting to detect FLAG-HA-tagged SCR product. (**E**) Luminescence-based SCR assay. The FLuc construct shown in (**C**) was transfected in HEK293 cells along with plasmids expressing dCas13 and *AGO1*-targeting or nontargeting gRNA. The luminescence was measured 48 h after transfection. FLuc activity relative to the activity of the co-transfected *Renilla* luciferase is shown. (**F**) Effect of CRISPR-dCas13 system on canonical translation and mRNA level. *AGO1*-3′UTR-*FLuc* construct without any stop codon in between was transfected in HEK293 cells along with plasmids expressing dCas13 and gRNA. Relative luciferase activity was measured as described above. qRT-PCR results in (**E**, **F**) show the expression of FLuc mRNA. Data information: Graphs in (**E**, **F**) are representatives of three independent experiments. Bars indicate mean ± SD ($N = 3$ Biological replicates). Two-sided Student's *t* test was used to calculate the *P* values. Source data are available online for this figure.

 © The Author(s)

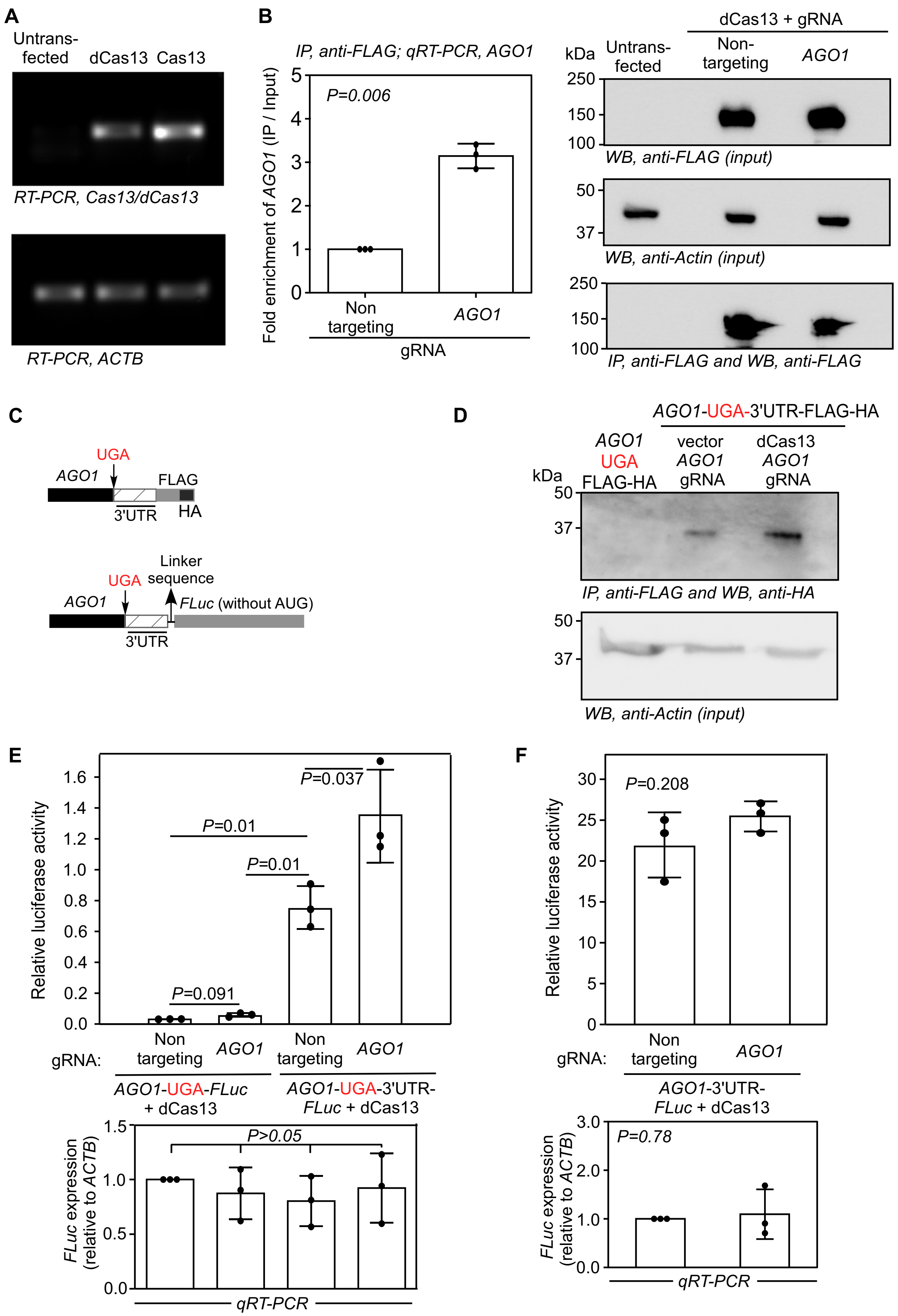
A
Untrans-fected
dCas13
Cas13
RT-PCR, Cas13/dCas13
RT-PCR, ACTB
B
IP, anti-FLAG; qRT-PCR, AGO1
P=0.006
Fold enrichment of AGO1 (IP / Input)
Non targeting
AGO1
gRNA
dCas13 + gRNA
kDa
Untrans-fected
Non-targeting
AGO1
WB, anti-FLAG (input)
WB, anti-Actin (input)
IP, anti-FLAG and WB, anti-FLAG
C
UGA
AGO1
FLAG
3'UTR
HA
Linker sequence
FLuc (without AUG)
D
AGO1-UGA-3'UTR-FLAG-HA
AGO1 UGA FLAG-HA
vector AGO1 gRNA
dCas13 AGO1 gRNA
IP, anti-FLAG and WB, anti-HA
WB, anti-Actin (input)
E
Relative luciferase activity
P=0.037
P=0.01
P=0.01
P=0.091
gRNA:
Non targeting
AGO1
AGO1-UGA-FLuc + dCas13
AGO1-UGA-3'UTR-FLuc + dCas13
FLuc expression (relative to ACTB)
P>0.05
qRT-PCR
F
P=0.208
AGO1-3'UTR-FLuc + dCas13
P=0.78

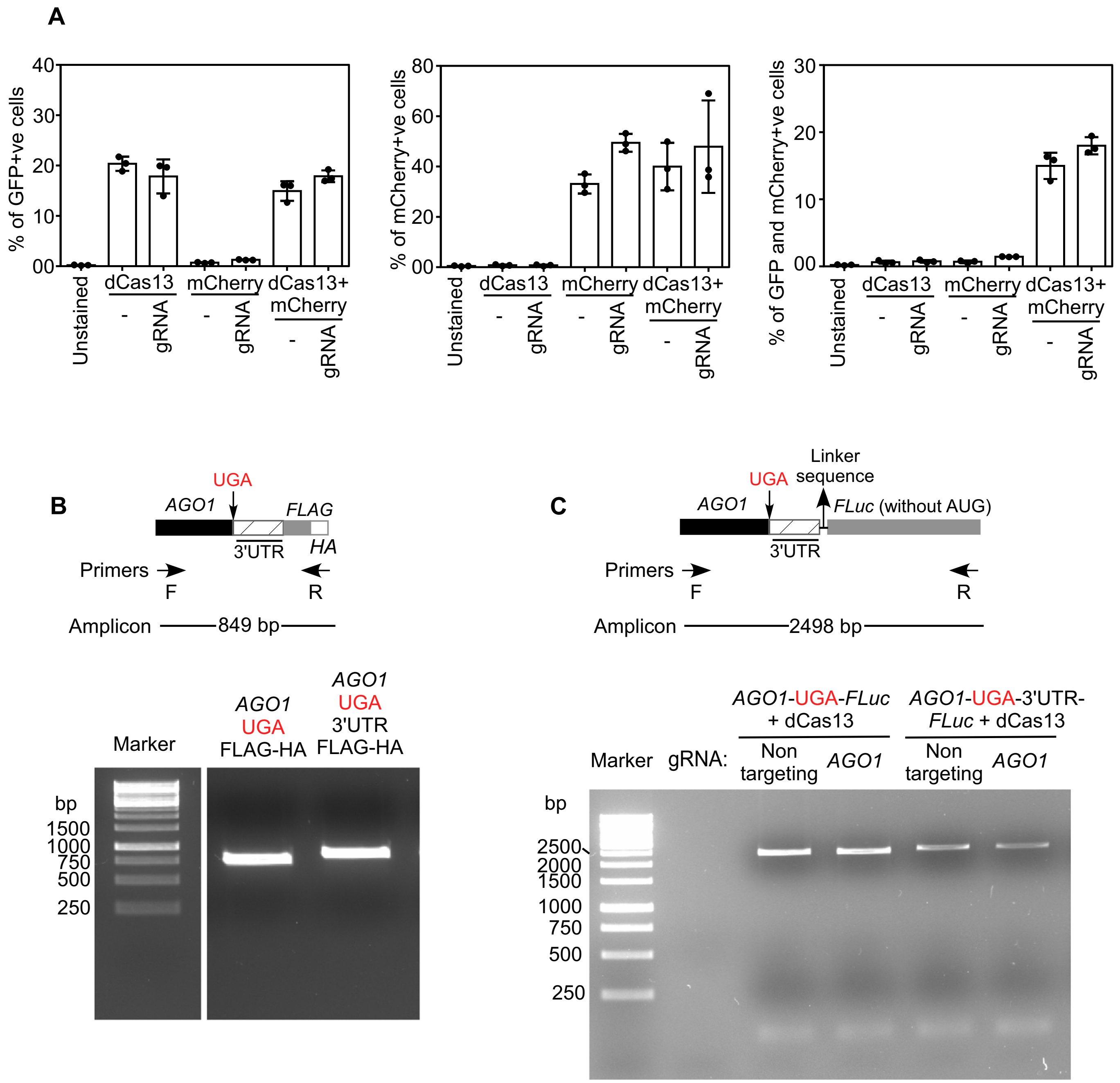

Figure EV2. (**A**) Evaluation of co-transfection efficiency. Constructs expressing dCas13 (also expresses green fluorescent protein), *AGO1*-targeting gRNA and mCherry (neutral reporter) were transfected in HEK293 cells. After 48 h, cells were subjected to flow cytometry analysis to quantify GFP+ve cells, mCherry+ve cells and double +ve cells. Graphs (mean ± SD, $N = 3$ biological replicates) are representatives of three independent experiments. (**B**, **C**) RT-PCR analysis of the transcripts expressed from the two reporter constructs used in SCR assays. Positions of the primers and the amplicon size are indicated. Following primers (5′ to 3′) were used for the RT-PCR: *AGO1*-HA: GTGCGGGTACAGCGACCACGGCAAGAG; TAGCGTAATCGGGCAC. AGO1-FLuc: GTGCGGGTACAGCGACCACGGCAAGAG; TTACAATTTGGACTTTCCG. Sequencing results of these transcripts are provided in Appendix Figs. S1 and S2. Source data are available online for this figure.

 © The Author(s)

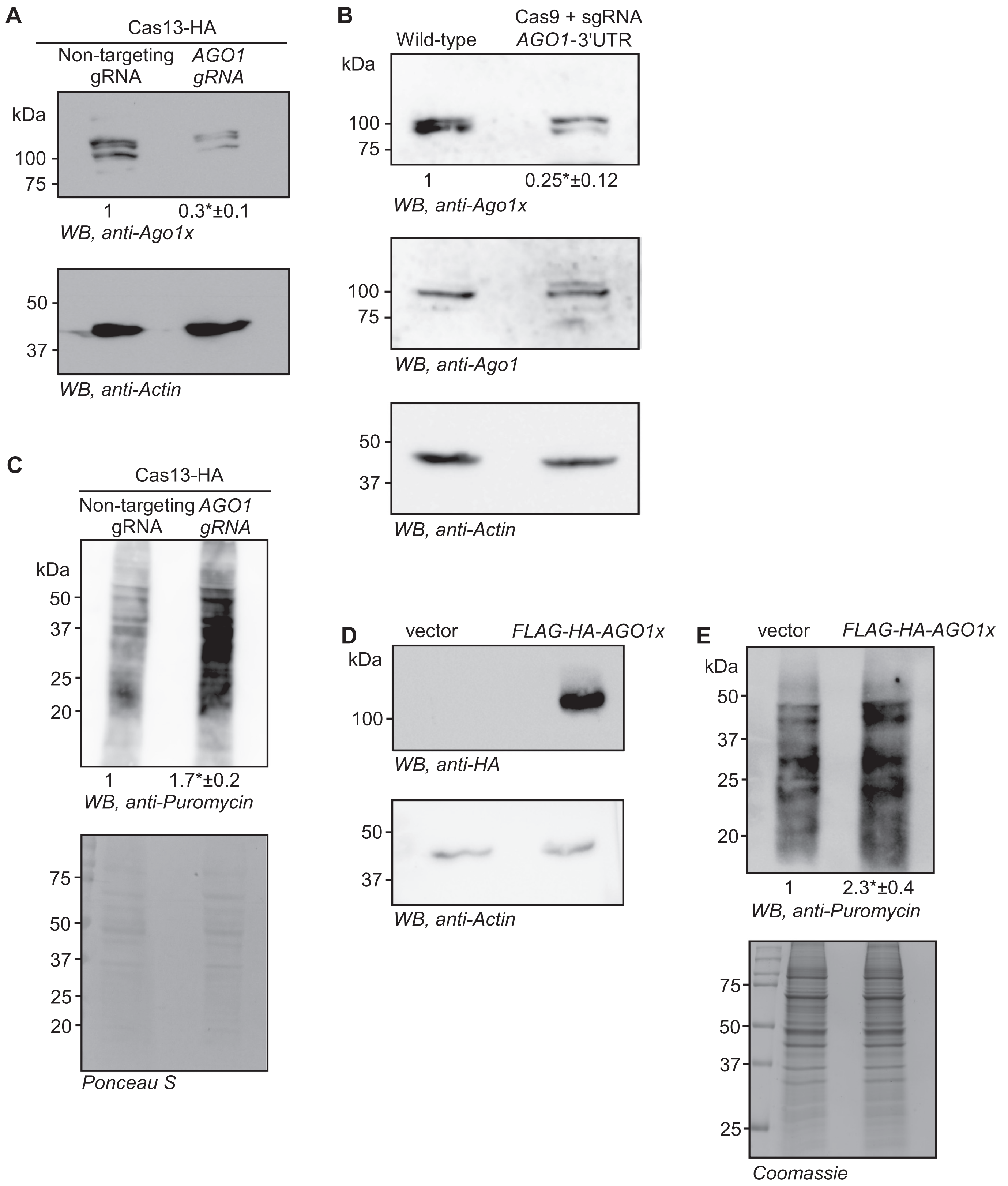
A
Cas13-HA
Non-targeting gRNA
AGO1 gRNA
kDa
100
75
1
0.3*±0.1
WB, anti-Ago1x
50
37
WB, anti-Actin
B
Wild-type
Cas9 + sgRNA AGO1-3'UTR
kDa
100
75
1
0.25*±0.12
WB, anti-Ago1x
100
75
WB, anti-Ago1
50
37
WB, anti-Actin
C
Cas13-HA
Non-targeting gRNA
AGO1 gRNA
kDa
50
37
25
20
1
1.7*±0.2
WB, anti-Puromycin
75
50
37
25
20
Ponceau S
D
vector
FLAG-HA-AGO1x
kDa
100
WB, anti-HA
50
37
WB, anti-Actin
E
vector
FLAG-HA-AGO1x
kDa
50
37
25
20
1
2.3*±0.4
WB, anti-Puromycin
75
50
37
25
Coomassie

Figure EV3. (**A**) Western blot showing reduced expression of Ago1x in HEK293 cells expressing Cas13 (catalytically active) and *AGO1*-targeting gRNA. Numbers indicate densitometry values (mean ± SD) from three experiments. $^*P = 0.011$, two-sided Student's *t* test. (**B**) Western blot showing expression of Ago1x and Ago1 in HeLa cells transfected with Cas9 and *AGO1*-3′UTR-targeting sgRNAs. Numbers indicate densitometry values (mean ± SD) from three experiments. $^*P = 0.008$, two-sided Student's *t* test. The sequencing of the genomic DNA PCR product revealed 51-nucleotide deletion in the proximal 3′UTR of *AGO1* (Appendix Fig. S3). (**C**) RiboPuromycylation assay performed in HEK293 cells expressing Cas13 (catalytically active) and *AGO1*-targeting gRNA. Numbers below indicate the densitometry values (mean ± SD, normalized to Ponceau staining) from three experiments. $^*P = 0.04$, two-sided Student's *t* test. (**D**) Western blot showing expression of exogenous FLAG-HA-tagged Ago1x in HEK293 cells. (**E**) RiboPuromycylation assay performed in HEK293 cells overexpressing FLAG-HA-tagged Ago1x in HEK293 cells. Numbers below indicate the densitometry values (mean ± SD, normalized to Coomassie staining) from three experiments. $^*P = 0.028$, two-sided Student's *t* test. Source data are available online for this figure.

© The Author(s)

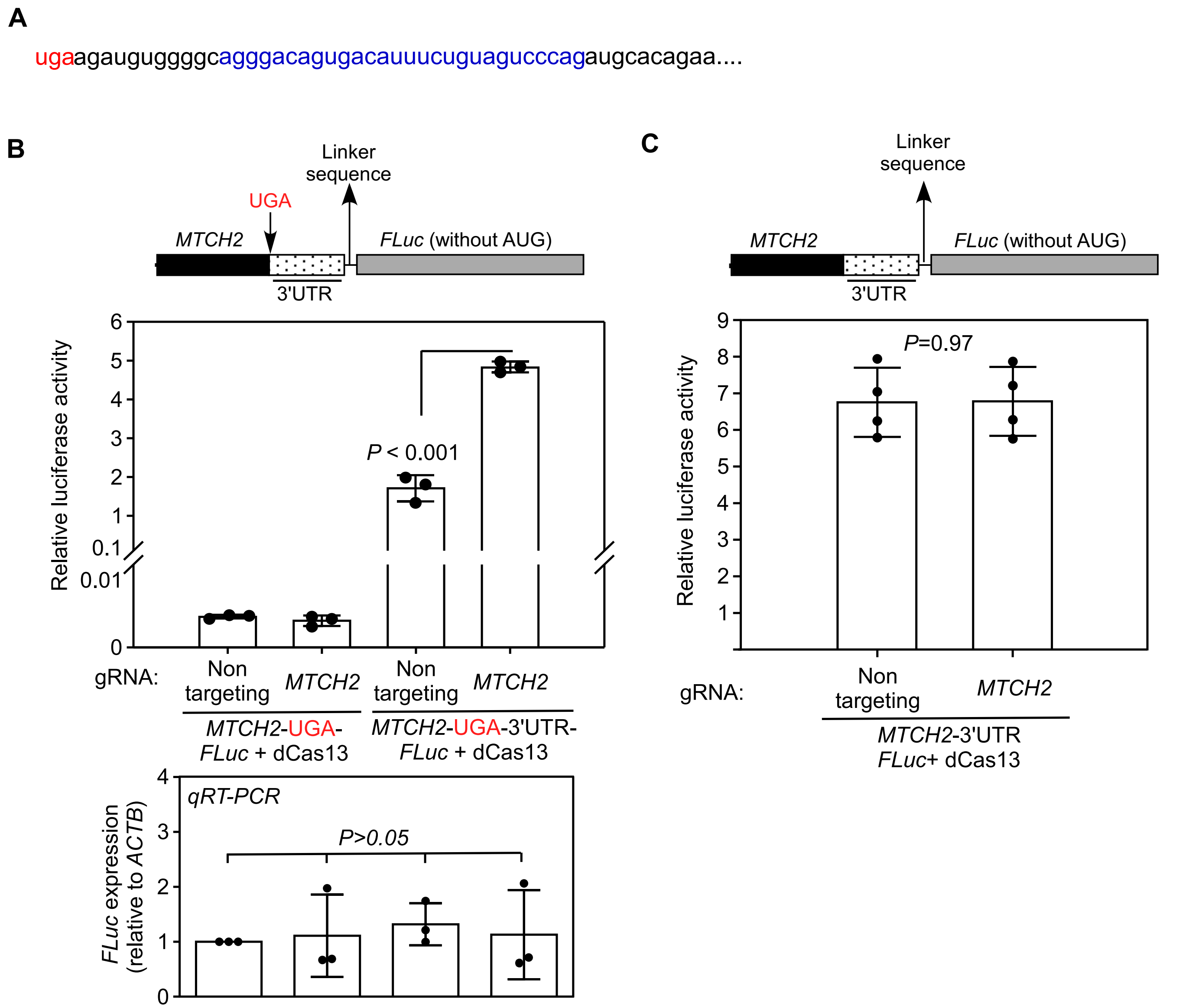

Figure EV4. Enhancement of SCR across the canonical stop codon of *MTCH2* using CRISPR-dCas13 system.

(**A**) The sequence of the proximal 3′UTR of *MTCH2*. The canonical stop codon (UGA) and the gRNA targeting region are in red and blue, respectively. (**B**) Luminescence-based SCR assay. The indicated constructs were transfected in HEK293 cells and firefly luciferase (FLuc) activity was measured 24 h after transfection. FLuc activity relative to the activity of the co-transfected *Renilla* luciferase is shown. Bottom panel shows the expression of *FLuc* mRNA measured by qRT-PCR. (**C**) Effect of CRISPR-dCas13 system on normal translation. *MTCH2*-3′UTR-FLuc construct without any stop codon in between was transfected in HEK293 cells along with plasmids expressing dCas13 and indicated gRNA. Relative luciferase activity was measured as described above. Data information: Graphs in (**B**, **C**) indicate mean ± SD, $N = 3$-4 experiments. Two-sided Student's t test was used to calculate the P value. Source data are available online for this figure.

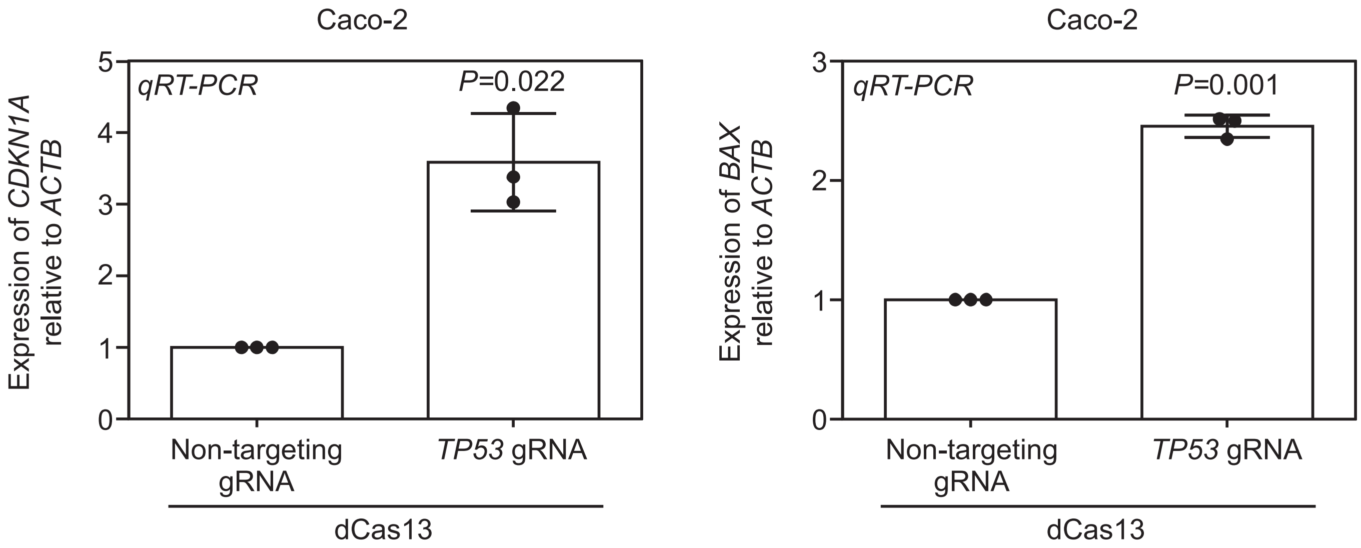

Figure EV5. qRT-PCR results showing the expression of *CDKN1A* (P21) and *BAX* in Caco-2 cells expressing dCas13 and the indicated gRNAs.

Graphs show mean ± SD ($N = 3$ technical replicates) and are representatives of three independent experiments. Two-sided Student's *t* test was used to calculate the *P* value. Following primers were used (5′ to 3′): *CDKN1A*: GGAAGACCATGTGGACCTGT and GGCGTTTGGAGTGGTAGAAA. *BAX*: GGGGACGAACTGGACAGTAA and CAGTTGAAGTTGCCGTCAGA. Source data are available online for this figure.

 © The Author(s)
